# Water electrolysis on La_1−*x*_Sr_*x*_CoO_3−*δ*_ perovskite electrocatalysts

**DOI:** 10.1038/ncomms11053

**Published:** 2016-03-23

**Authors:** J. Tyler Mefford, Xi Rong, Artem M. Abakumov, William G. Hardin, Sheng Dai, Alexie M. Kolpak, Keith P. Johnston, Keith J. Stevenson

**Affiliations:** 1 Department of Chemistry, The University of Texas at Austin, Austin, Texas 78712, USA; 2 Department of Mechanical Engineering, Massachusetts Institute of Technology, Cambridge, Massachusetts 02139, USA; 3 Center for Electrochemical Energy Storage, Skolkovo Institute of Science and Technology, 143026 Moscow, Russia; 4 Electron Microscopy for Material Science, University of Antwerp, Groenenborgerlaan 171, B-2020 Antwerp, Belgium; 5 Texas Materials Institute, The University of Texas at Austin, Austin, Texas 78712, USA; 6 Chemical Sciences Division, Oak Ridge National Laboratory, Oak Ridge, Tennessee 37831, USA; 7 Department of Chemical Engineering, The University of Texas at Austin, Austin, Texas 78712, USA; 8 Center for Nano and Molecular Science and Technology, The University of Texas at Austin, Austin, Texas 78712, USA

## Abstract

Perovskite oxides are attractive candidates as catalysts for the electrolysis of water in alkaline energy storage and conversion systems. However, the rational design of active catalysts has been hampered by the lack of understanding of the mechanism of water electrolysis on perovskite surfaces. Key parameters that have been overlooked include the role of oxygen vacancies, B–O bond covalency, and redox activity of lattice oxygen species. Here we present a series of cobaltite perovskites where the covalency of the Co–O bond and the concentration of oxygen vacancies are controlled through Sr^2+^ substitution into La_1−*x*_Sr_*x*_CoO_3−*δ*_. We attempt to rationalize the high activities of La_1−*x*_Sr_*x*_CoO_3−*δ*_ through the electronic structure and participation of lattice oxygen in the mechanism of water electrolysis as revealed through *ab initio* modelling. Using this approach, we report a material, SrCoO_2.7_, with a high, room temperature-specific activity and mass activity towards alkaline water electrolysis.

## Abstract (web summary)

Perovskite oxides are attractive candidates as catalysts for water electrolysis however their rational design is rare. Here, the authors report a series of cobaltite perovskites where the covalency of the Co-O bond and concentration of oxygen vacancies are controlled, and assess their catalytic performance.

## Graphical abstract

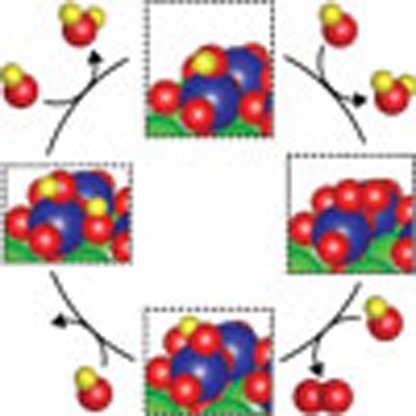

The scarcity of fossil fuels and the increasing awareness of the environmental and geopolitical problems associated with their use have encouraged significant efforts towards the development of advanced energy storage and conversion systems using materials that are cheap, abundant and environmentally benign. A major thrust in the field of renewable energy has been to develop higher power and more energy-dense storage devices, including low-temperature regenerative fuel cells and rechargeable metal–air batteries that function through the electrocatalysis of oxygen. Inherent to these systems are the electrolysis of water (2H_2_O→O_2_+4H^+^+4e^−^; oxygen evolution reaction (OER)) and the reduction of molecular oxygen (O_2_+4H^+^+4e^−^→2H_2_O; oxygen reduction reaction (ORR)), both of which require the use of an electrocatalyst due to their slow reaction kinetics. The most active catalysts for the ORR are Pt-alloys and other precious metals, Ir, Ru and Pd^1^,^2^,^3^. However, while the Pt group metals perform well for the ORR, the formation of an oxide surface film at high potentials, especially in the case of Pt, decreases their ability to catalyse the OER^4^. This problem, coupled with the Pt group metal scarcity and restrictive cost represent major roadblocks to mass adoption of fuel cells and metal–air batteries in renewable energy technologies.

Using alkaline electrolytes opens up the possibility to use transition metal oxides as catalysts due to their structural stability, resistance to electrolytic corrosion and their high activities for both the OER and ORR^5^,^6^,^7^. Among the wide variety of metal oxides available, the crystal family of perovskite oxides ABO_3±*δ*_, of which A is a rare-earth or alkaline earth element and B is a transition metal, are attractive candidates due to their high ionic and electronic conductivities, structural stability, and the ability to substitute into the A and B sites elements of varying valency, electronegativity or ionic size to tune the structural, physical and electronic properties of the catalyst. Even though the electrolysis of water to oxygen is one of the most extensively studied reactions, predating even the fields of catalysis and electrochemistry, the lack of a conclusive mechanism for metal oxides in alkaline electrolyte remains a significant limitation in the rational design of electrocatalysts for the OER^8^. Thus, much of the research on perovskites for the OER and ORR has been focused on identifying descriptors for the activities of perovskites based on the electronic and structural properties of the surface or bulk^9^,^10^,^11^. Since the initial discovery of La_0.8_Sr_0.2_CoO_3_ as an active ORR catalyst, many mechanistic theories have been put forward over the past 40 years^12^. A recent review summarizes the current understanding of mechanistic processes for the OER, specifically highlighting correlations between bulk and surface properties of metal oxides and their electrocatalytic activities^13^. Notably, the idea that the e_g_ filling of the transition metal in the ABO_3_ perovskite controls the intermediates binding strength and thus the electrocatalytic activity has recently gained significant credence^14^,^15^. However, we have observed that among a series of perovskites with a nominal e_g_ filling of ∼1 (LaBO_3_, where B=Mn, Co, Ni, or Ni_0.75_Fe_0.25_), there exists significant differences in their activities for both the ORR and the OER, indicating that the surface chemistry may not be adequately rationalized by bulk electronic descriptions^16^,^17^.

A previously overlooked parameter concerns the role of oxygen vacancy defects, which allows for crystalline oxygen to be mobile at the surface of perovskites. It is well-known that the stoichiometry of oxygen in the crystal structure of perovskites often differs from the nominal value of 3 for the formula ABO_3_, affecting both the lability of surface oxygen and reflecting the underlying electronic structure of these materials^18^,^19^,^20^. The degree of vacancy formation reflects the relative positions of the transition metal 3d bands compared with the oxygen 2p band in the crystal, with more covalent systems exhibiting higher vacancy concentrations as shown in Fig. 1. In addition, it is well-documented that the concentrations of oxygen vacancies in perovskite electrodes can be controlled through an applied electrical potential, with room temperature diffusion coefficients of lattice oxygen for a number of perovskites in the range of 10^−14^ to 10^−11^ cm^2^ s^−1^ (refs 21, 22, 23, 24, 25, 26). In a previous paper, we demonstrated that this effect could be used as a means of pseudocapacitive energy storage in an oxygen-deficient LaMnO_2.91_ electrode^27^. We have previously hypothesized the role of lattice oxygen and vacancy exchange in the OER mechanism on LaNiO_3_ refs 16, 17. We now revisit this idea to investigate the role of mobile lattice oxygen in the electrolysis of water by examining the system La_1−*x*_Sr_*x*_CoO_3−*δ*_, 0≤*x*≤1. Through substitution of the lower valence Sr^2+^ ion for La^3+^, the amount of oxygen vacancy defects and the oxidation state of cobalt can be tuned through the relation^28^:


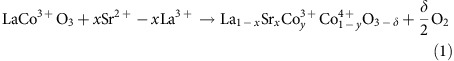


where, *δ* is the oxygen non-stoichiometry parameter, *x* is the amount of Sr^2+^, and *y* is the amount of Co^3+^ in La_1−*x*_Sr_*x*_CoO_3−*δ*_, hereafter referred to as LSCO(1−*x*)*x* (that is, LSCO28 for La_0.2_Sr_0.8_CoO_3−*δ*_).

Herein, we describe the intrinsic activities of La_1−*x*_Sr_*x*_CoO_3−*δ*_ for the OER across the full series from 0≤*x*≤1, including the previously unreported perovskite phase SrCoO_2.7_ with the layered ordering of oxygen vacancies. The controlled substitution of Sr^2+^ for La^3+^ across the full phase space of the LSCO system while maintaining the perovskite structure allows us to probe the effects of covalency, vacancy defects and oxygen exchange on the electrocatalysis of the OER. The high activities for materials with *x*>0.4 are rationalized through the high oxygen ion diffusivity and the covalency of the Co 3d and O 2p bonding in these materials allowing access to a newly hypothesized lattice oxygen-based mechanism as predicted through DFT modelling.

## Results

### Crystallographic characterization

LCO, LSCO and SCO samples were synthesized using our previously developed reverse-phase hydrolysis scheme, using a 950 °C calcination temperature instead of 700 °C to ensure that the correct phase was synthesized^16^,^17^,^27^. Figure 2a shows the powder X-ray diffraction patterns for the system, demonstrating the successful synthesis of the perovskite phases across the whole-composition range. The only minor admixture found in the LCO and LSCO samples was Co_3_O_4_. The crystal structures of all compositions have been verified using a combination of powder X-ray diffraction and transmission electron microscopy. The unit cell parameters and space groups of the respective materials are given in Supplementary Table 1. The powder X-ray diffraction and selected area electron diffraction (SAED) patterns of the *x*=0–0.4 compositions are characteristic of the perovskite 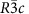 structure with the *a*^−^*a*^−^*a*^−^ tilting distortion of the octahedral framework (Fig. 2b,e). The monoclinic distortion due to orbital ordering reported for this compositional range was not detected being beyond resolution of our powder X-ray diffraction experiment^29^,^30^,^31^. The LSCO46 composition crystallizes in a cubic 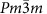 perovskite structure. In the crystal structures of LSCO28 and SCO ordering of oxygen vacancies becomes obvious from both SAED patterns and high-angle annular dark-field scanning transmission electron microcopy (HAADF-STEM) images (Fig. 2c,d,f,g). Oxygen vacancies reside in the (CoO_2-δ_) anion-deficient perovskite layers alternating with the complete (CoO_2_) layers that results in a tetragonal *a*_p_ × *a*_p_ × 2*a*_p_ (*a*_*p*_ indicates the parameter of the perovskite subcell) supercell in LSCO28. The anion-deficient layers manifest themselves as faintly darker stripes in the HAADF-STEM images (marked with arrowheads in Fig. 2f,g), which according to Kim *et al*.^32^ is related to the structural relaxation in these planes. The anion-deficient layers form nanoscale-twinned patterns in both the LSCO28 and SCO samples (Fig. 2f,g). In general, the crystallographic observations on the LCO and LSCO samples are in agreement with the La_1−*x*_Sr_*x*_CoO_3−*δ*_ phase diagram^33^. However, in contrast to the earlier reported Sr_2_Co_2_O_5_ brownmillerite or hexagonal Sr_6_Co_5_O_15_ phases^34^,^35^, the SCO sample demonstrates another type of oxygen vacancy ordering. The [010]_p_ SAED pattern of SCO (Fig. 2d, top) is strongly reminiscent to that of the *Ln*_1−*x*_Sr_*x*_CoO_3−*δ*_ (*Ln*=Sm-Yb, Y) perovskites with the *I*4/*mmm* 2*a*_p_ × 2*a*_p_ × 4*a*_p_ supercell^33^,^36^,^37^. A detailed deconvolution of this SAED pattern into contributions from the twinned domains is presented in Supplementary Fig. 1. This supercell also allows complete indexing of the powder X-ray diffraction pattern of SCO (Supplementary Fig. 2). The layered ordering of the oxygen vacancies in the LSCO28 and SCO samples was directly visualized using annular bright-field STEM (ABF-STEM) imaging (Fig. 3a,b). In both structures the anion-complete (CoO_2_) and anion-deficient (CoO_2−δ_) layers can be clearly distinguished, alternating along the *c*-axis of the tetragonal supercells. However, establishing the exact ordering patterns of the oxygen atoms and vacancies in these (CoO_2−δ_) layers requires more detailed neutron powder diffraction investigation.

In order to understand the effects of Sr^2+^ substitution on oxygen vacancy concentrations in La_1−*x*_Sr_*x*_CoO_3−*δ*_, iodometric titrations were performed. It should be noted that processing conditions affect the oxygen content and oxidation state of cobalt significantly through equation 1. The results of the iodometric titrations are presented in Table 1. As can be seen, there is both an increase in the bulk oxidation state of Co as well as an increase in the concentration of oxygen vacancies as lower valence Sr^2+^ is substituted for La^3+^. The high concentration of oxygen vacancies in SrCoO_2.7_ corroborates their pronounced layered ordering.

### Microstructural characterization

The overall morphology of the LSCO series was investigated with bright-field TEM images, presented in Supplementary Fig. 3. The samples consist of highly agglomerated and partially sintered nanoparticles with size ranging from 20–50 nm to few hundred nanometres. The LCO and SCO materials demonstrate somewhat larger and more sintered crystallites compared with those of the mixed LSCO samples. HAADF-STEM and ABF-STEM images of the surface structure of LCO and SCO are shown in Supplementary Fig. 4, where the particles remain crystalline at the surface and for SCO the anion-deficient layers, evident through the nanoscale-twinned domain columns, extend to the surface. Brunauer–Emmett–Teller surface areas measured through N_2_ adsorption showed similar surface areas for all samples of 3.1–4.5 m^2^ g^−1^ (Supplementary Table 2). This surface area is approximately half the surface area of the materials reported in our previous studies, which results from the higher calcination temperatures used for the LSCO series than the previously investigated LaCoO_3_, LaNiO_3_, LaMnO_3_ and LaNi_0.75_Fe_0.25_O_3_.

### Electrochemical characterization

In order to better understand the role of oxygen vacancies in La_1−*x*_Sr_*x*_CoO_3−*δ*_ during electrochemical applications, the intercalation of oxygen in LSCO was studied using cyclic voltammetry in Ar saturated 1 M KOH solutions. The insertion and removal of oxygen ions appear as redox peaks in Fig. 4a. It is apparent that an increase in the oxygen vacancy concentration as Sr^2+^ is substituted for La^3+^ in LSCO increases the tendency for oxygen intercalation as indicated through the high current densities measured in the intercalation region. In addition, it is interesting to note that the position of the intercalation redox peaks shifts to higher potentials with increased oxygen vacancies which can be described through the common pseudocapacitive Nernst Equation:


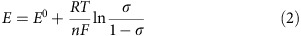


where, *E* represents the measured potential for oxygen intercalation, *E*^*0*^ represents the standard potential for oxygen intercalation, *R* is the universal gas constant (8.3145 J K^−1^ mol^−1^), *T* is the temperature during the measurement, *F* is Faraday's constant (96,485 C mol^−1^), and *σ* is the occupancy fraction of accessible lattice vacancy sites^38^ for the reaction:


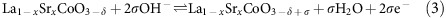


This type of Nernst Equation is commonly associated with pseudocapacitive-type intercalation mechanisms, indicative of facile oxygen ion diffusion.

The diffusion rates of oxygen ions in LSCO were measured chronoamperometrically based on a bounded 3D solid-state diffusion model with a rotating disk electrode (RRDE) rotating at 1,600 r.p.m. in Ar saturated 1 M KOH^39^,^40^,^41^. These results are presented in Fig. 4b, and a more detailed description of the theory behind the model is included as Supplementary Fig. 5. It was found that SCO, with a vacancy concentration of *δ*=0.30±0.03, had a diffusion rate of D=1.2±0.1 × 10^−12^ cm^2^ s^−1^ at room temperature, which is ∼40 × faster than for LCO, with a complete oxygen sublattice and a diffusion rate of D=3±1 × 10^−14^ cm^2^ s^−1^. As a general comment, diffusion coefficients in the range of 10^−9^ to 10^−14^ cm^2^ s^−1^ have been found as usual values for the short circuit diffusion of oxygen along high-diffusivity pathways, including grain boundaries^24^. Although it is unclear whether the measured diffusion rates are from bulk diffusion or along grain boundaries, isotope tracer studies have shown that diffusion rates trend in the order of surface oxygen>oxygen at grain boundaries>bulk oxygen in perovskite systems, and thus the fast diffusion rates found in this study represent the lower boundary on the mobility of oxygen at the surface^42^. Further, the crystallite size and density of grain boundaries is relatively consistent across the LSCO series due to the similar synthetic conditions, indicating that the diffusion rates can at least be compared against each other. The results indicate that the diffusion rates scale with Sr concentration because of the correlation with vacancies and Sr content. The results highlight the benefit of substitution of a lower valence ion into the A-site as an effective means of increasing the mobility of oxygen in perovskite oxide electrodes.

### The electrolysis of water

The OER activities for LSCO and for a commercial IrO_2_ sample were quantified through cyclic voltammetry in O_2_ saturated 0.1 M KOH at 1,600 r.p.m., as shown in Fig. 5a. Each material was mixed at a mass loading of 30 wt% perovskite on a mesoporous nitrogen-doped carbon (NC) or onto Vulcan Carbon XC-72 (VC) for stability measurements. An evaluation of the carbon loading and total mass loading is presented in Supplementary Fig. 6, Supplementary Table 5 and the Supplementary Discussion. There is a shift towards more active Tafel slopes with increasing Sr content, with LCO and IrO_2_ having similar Tafel slopes of *∂* *V*/*∂* ln *i*=58 mV dec^−1^ (≈2*RT*/*F*) which decreases towards SCO with a Tafel slope of *∂* *V*/*∂* ln *i*=31 mV dec^−1^ (≈*RT/F*). This shift of Tafel slope for the OER may be indicative of the facile surface kinetics for oxygen exchange with increasing vacancy content, whereby OER kinetics that are limited by high-coverage Langmuir like behaviour where surface oxygen is not exchanged rapidly (*θ*→1) show Tafel slopes of 2*RT/F*. In contrast, those materials showing more rapid surface oxygen exchange in the intermediate coverage Temkin condition range (0.2<*θ*<0.8) have slopes of *RT/F*^9^. The specific activities at an overpotential of 400 mV, based on perovskite surface area from BET, are presented in Fig. 5b. It is clear that substitution of Sr^2+^ for La^3+^ in LSCO, and thereby the creation of oxygen vacancies, is beneficial to the OER, with the fully substituted SrCoO_2.7_ at 28.4 mA cm^−2^_ox_ which is ∼6 × more active than LaCoO_3.005_ (4.3 mA cm^−2^_ox_), ∼23 × more active than the commercial IrO_2_ sample (1.2 mA cm^−2^_ox_), and ∼1.5 × more active than previously reported high-vacancy concentration cobaltite perovskites (Ba_0.5_Sr_0.5_Co_0.8_Fe_0.2_O_2.6_: ∼20 mA cm^−2^_ox_; Pr_0.5_Ba_0.5_CoO_2.85_: ∼20 mA cm^−2^_ox_) (refs 14, 43). In addition, due to the small particle size from the reverse-phase hydrolysis synthesis, SrCoO_2.7_ (3.6 m^2^ g^−1^) had a mass activity of 1,020±20 mA mg^−1^_ox_ at +1.63 V versus the reversible hydrogen electrode (RHE), which is ∼2 × more active than BSCF with a similar surface area (∼500 mA mg^−1^_ox_, 3.9 m^2^ g^−1^) (ref. 14). To verify that the measured current was due only to the OER, and not to side-reactions or corrosion of the electrode material, rotating-ring-disk (RRDE) cyclic voltammetry was performed with a Pt ring poised at +0.4 V versus RHE, whereby O_2_ generated at the disk from the OER is collected and reduced at the ring. The results for SrCoO_2.7_/NC and IrO_2_/NC are shown in Fig. 5c. The collection efficiency for both SrCoO_2.7_/NC and IrO_2_/NC was 37%, which was equal to the collection efficiency measured during calibration of the RRDE for the oxidation of 0.3 mM ferrocene-methanol in 0.1 M KCl. Therefore, we can confirm that the current is exclusively due to the generation of oxygen on the SCO or the IrO_2_ surface within the precision of the RRDE measurements.

The stability of SrCoO_2.7_ and of the carbon supports under OER conditions were tested galvanostatically at 10 A g^−1^_ox_ and 1,600 r.p.m., shown in Fig. 5d. As is readily apparent, both the NC and VC are not stable carbon supports for the OER, and we hypothesize that this dominates the mechanism of failure for the composite electrodes at potentials >+1.65 V versus RHE. However, other variables may be responsible for the failure of the electrodes, including the degradation of the Nafion binder due to the oxidative conditions and the rapid rotation of the electrode. SrCoO_2.7_, however, appears to be active enough to sustain the OER for 24 h at 10 A g^−1^_ox_ without reaching the potential where rapid carbon corrosion occurs. Further studies are needed in order to better understand the variables that influence catalyst stability, however, it is clear that carbon may not be the optimal catalyst support under the OER conditions. In addition, it should be noted that IrO_2_ which has become the benchmark comparison for OER catalysts is not stable under the anodic conditions of the OER, forming the soluble complex anion IrO_4_^2−^ in alkaline environments^44^. This is demonstrated in the stability plot in Fig. 5d, where even the unsupported IrO_2_ electrode failed after ∼14 h.

**Figure 1:**
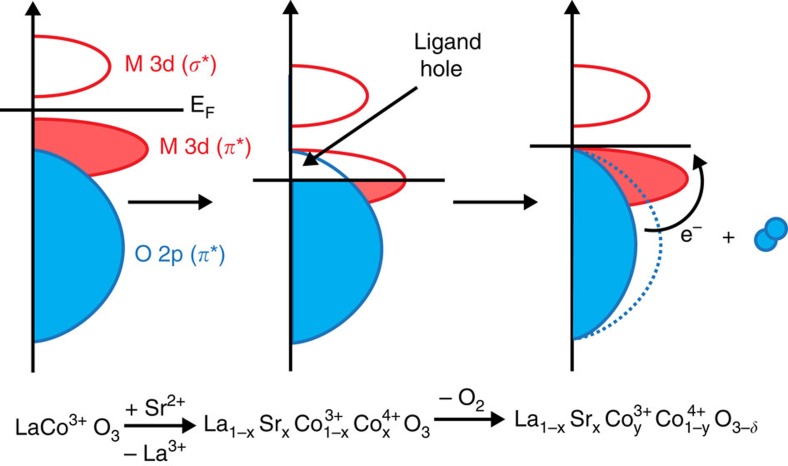
Relationship between oxygen vacancy concentration and Co–O bond covalency. As the oxidation state of Co is increased through Sr^2+^ substitution, the Co 3d/O 2p band overlap is increased (covalency increases) and the Fermi level decreases into the Co 3d/O 2p π* band, creating ligand holes. Oxygen is released from the system resulting in oxygen vacancies and pinning the Fermi level at the top of the Co 3d/O 2p π* band^61^.

**Figure 2:**
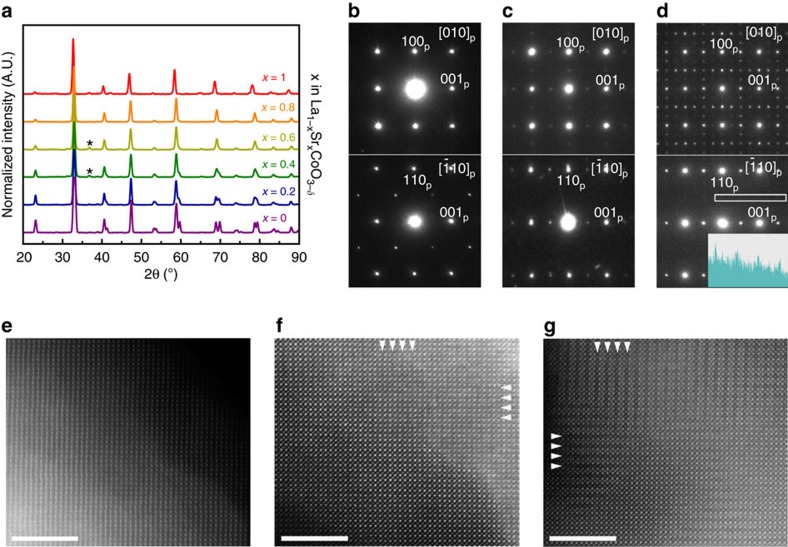
Structural characterization of La_1−*x*_Sr_*x*_CoO_3−*δ*_. (**a**) Powder X-ray diffraction patterns for La_1−*x*_Sr_*x*_CoO_3−*δ*_ (0≤*x*≤1). The reflection from Co_3_O_4_ is marked with an asterisk. (**b**–**d**) SAED patterns of LSCO82 (**b**), LSCO28 (**c**) and SCO (**d**). The reflections of the basic perovskite structure are indexed. The [−110]_p_ SAED pattern of LSCO82 shows weak G_p_±1/2<111>_p_-type reflections (G_p_—reciprocal lattice vector of the perovskite structure) characteristic of the *a*^−^*a*^−^*a*^−^ octahedral tilting distortion of the perovskite structure. The [010]_p_ SAED pattern of LSCO28 demonstrates the orientationally twinned G_p_±1/2<001>_p_ superlattice reflections resulting in the *P*4/*mmm a*_p_ × *a*_p_ × 2*a*_p_ supercell. The superstructure in the [010]_p_ SAED pattern of SCO can be described with the G_p_±*n*/4<201>_p_ (*n*—integer) and G_p_±1/2<110>_p_ superstructure vectors corresponding to the orientationally twinned *I*4/*mmm* 2*a*_p_ × 2*a*_p_ × 4*a*_p_ supercell (see details in Supplementary Fig. 1). Note that the G_p_±1/2<110>_p_ superlattice reflections are barely visible in the 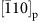 SAED patterns of SCO, but the intensity profile (shown as insert in **d**) along the area marked with the white rectangle demonstrates their presence undoubtedly. (**e**–**g**) [010]_p_ HAADF-STEM images of LSCO82 (**e**), LSCO28 (**f**) and SCO (**g**). The image of LSCO82 shows uniform perovskite structure, whereas the images of LSCO28 and SCO show faint darker stripes spaced by 2*a*_p_ (marked by arrowheads) indicating nanoscale-twinned arrangement of the alternating (CoO_2_) perovskite layers and (CoO_2−δ_) anion-deficient layers. Scale bars are 5 nm.

**Figure 3:**
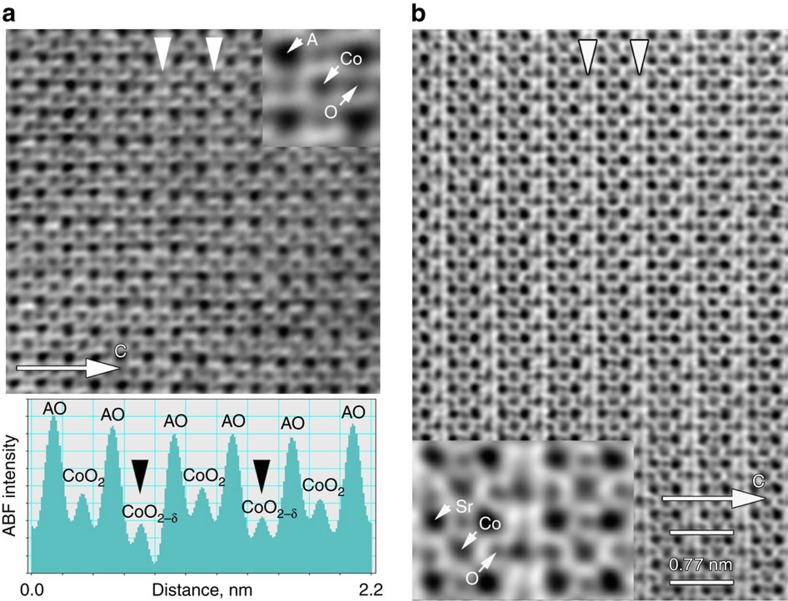
ABF-STEM imaging of oxygen vacancy ordering in La_1−*x*_Sr_*x*_CoO_3−*δ*_ (*x*=0.8, 1.0). (**a**) [001]_p_ ABF-STEM image of LSCO28 showing the cation and anion sublattices. The contrast is inverted in comparison with the HAADF-STEM images. The assignment of the atomic columns is shown in the enlargement at the top right corner. Half of the perovskite (CoO_2_) layers appear brighter indicating oxygen deficiency (marked with white arrowheads). The complete (CoO_2_) layers and anion-deficient (CoO_2−*δ*_) layer alternate (see the ABF intensity profile below, the anion-deficient layers are marked with black arrowheads) resulting in doubling of the perovskite lattice parameter in the direction perpendicular to the layers. (**b**) [001]_P_ ABF-STEM image of SCO showing layered anion-vacancy ordering. The (CoO_2−*δ*_) layers are marked with the white arrowheads and demonstrate the contrast clearly distinct from that of the (CoO_2_) layers. The assignment of the atomic columns is shown in the enlarged part at the bottom left.

**Figure 4:**
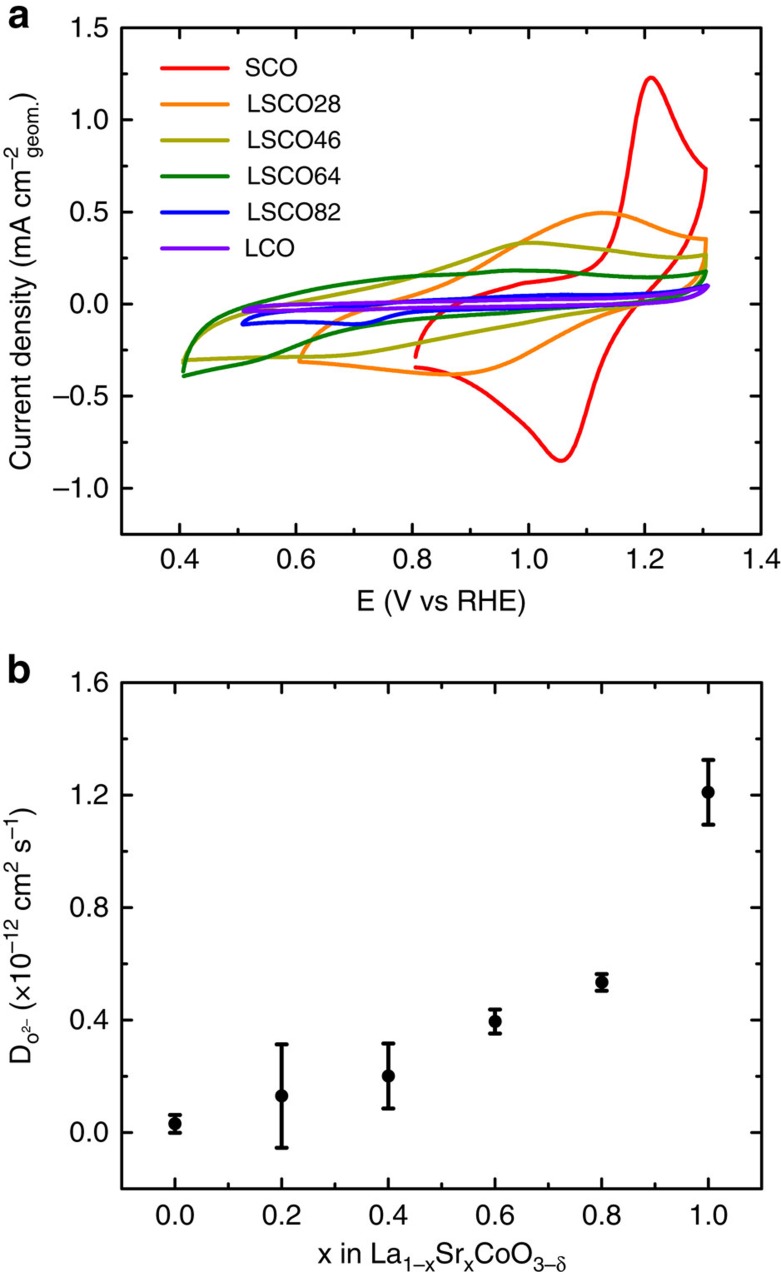
Electrochemical oxygen intercalation into La_1−*x*_Sr_*x*_CoO_3−*δ*_. (**a**) Cyclic voltammetry at 20 mV s^−1^ for each member of LSCO in Ar saturated 1 M KOH. The redox peaks, indicative of the insertion and removal of oxygen from the crystal, shift to higher potentials with increasing Sr^2+^ and oxygen vacancy concentrations. (**b**) Oxygen diffusion rates measured at 25 °C chronoamperometrically. The diffusion rate increases with Sr^2+^ and oxygen vacancy concentrations as well. Error bars represent the standard deviation of triplicate measurements.

**Figure 5:**
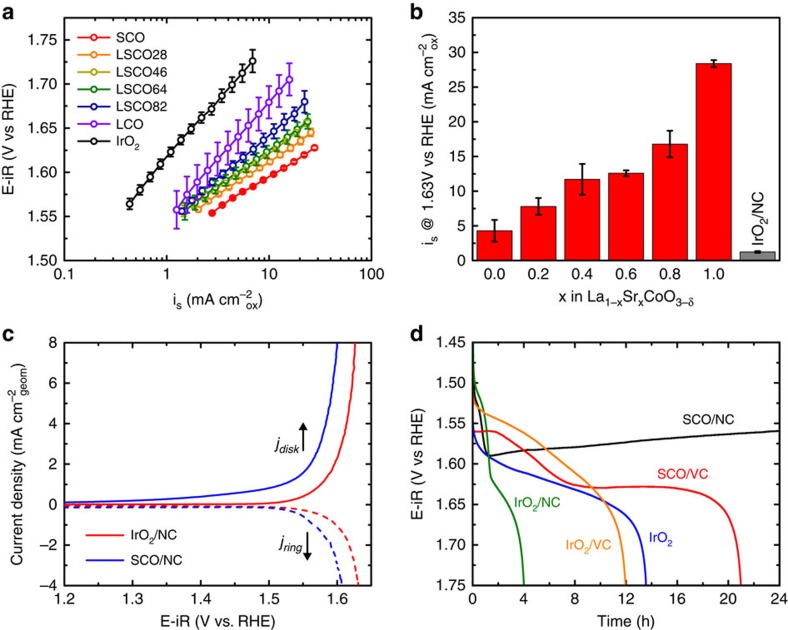
Electrochemical characterization of La_1−*x*_Sr_*x*_CoO_3−*δ*_ for the OER. (**a**) Capacitance corrected specific OER current densities in O_2_ saturated 0.1 M KOH, a scan rate of 10 mV s^−1^, and *ω*=1,600 r.p.m., for 30 wt% La_1−*x*_Sr_*x*_CoO_3−*δ*_ supported on 2 at. % NC. The performance of 30 wt% IrO_2_ supported on 2 at. % NC is included as a reference. (**b**) Specific activities of La_1−*x*_Sr_*x*_CoO_3−*δ*_ and IrO_2_ at a 400 mV overpotential for the OER (1.63 V versus RHE). (**c**) Confirmation of oxygen generation using a RRDE. The disk has a thin layer of either 30 wt% SrCoO_2.7_/NC or 30 wt% IrO_2_/NC and the ring is Pt. O_2_ is generated at the disk then reduced back to OH^−^ at the ring which is poised at +0.4 V versus RHE. The collection efficiency of the RRDE was found to be 37%. (**d**) Galvanostatic stability at 10 A g^−1^_ox_ and *ω*=1,600 r.p.m. of SrCoO_2.7_ and IrO_2_ supported on two different carbons, 2 at% nitrogen-doped NC and non-nitrogen doped VC. It is evident that both carbons are unstable at the anodic potentials of the OER, with rapid degradation occurring for all samples once the potential is >1.65 V versus RHE. The high activity and stability of SrCoO_2.7_ on NC allows the electrode to generate 10 A g^−1^_ox_ of current without reaching this potential, which results in a relatively stable catalyst for 24 h of operation. For all electrochemical studies the mass loading of the electrode was 51 μg_tot_ cm^−2^_geom_. Error bars represent the standard deviation of triplicate measurements.

**Figure 6:**
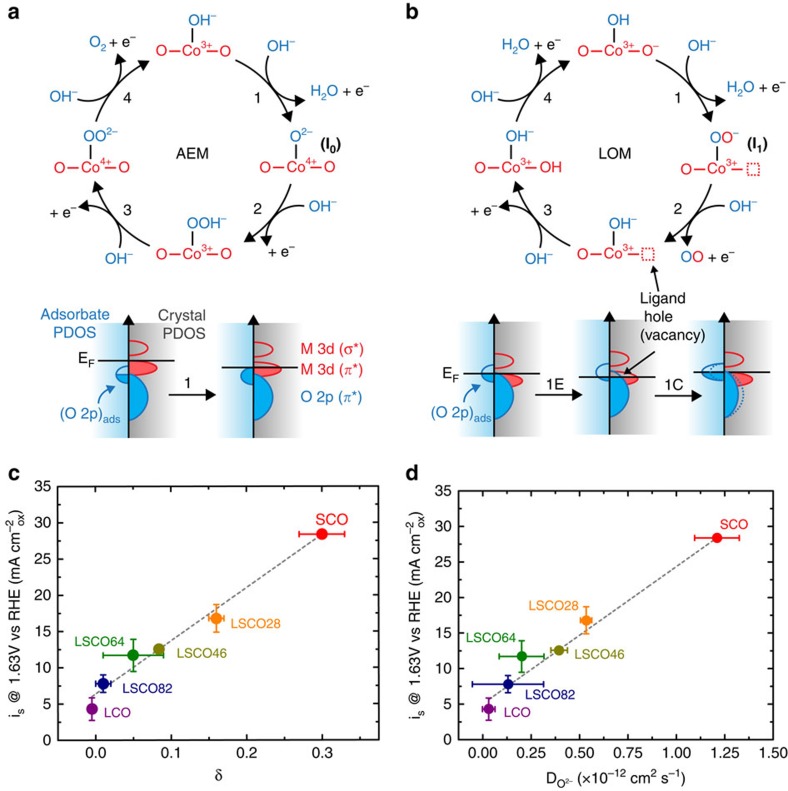
Oxygen evolution mechanisms on La_1−*x*_Sr_*x*_CoO_3−*δ*_ and activity correlations. (**a**) AEM^14^,^62^. In the AEM, the transition metal 3d bands are significantly higher in energy than the O 2p band in the lattice as shown qualitatively in the PDOS diagram below the mechanism. Because of this, all intermediates during the reaction originate from the electrolyte and Co in the active-site undergoes the catalytic redox reactions. This allows Co to access a higher oxidation state of Co^4+^ in Step 1 (**a**) AEM. As the covalency of the material increases, the transition metal 3d bands are lowered into the O 2p band in the lattice, where the Fermi energy is pinned at the top of the O 2p band through generation of oxygen vacancies^61^. In contrast, in Step 1 (**b**) of the LOM, applying an anodic potential oxidizes a ligand hole in the O 2p band allowing for exchange of lattice oxygen to the adsorbed intermediate to yield the superoxide ion O_2_^−^ rather than oxidizing Co to Co^4+^. This is shown qualitatively in the PDOS diagram below the mechanism where Step 1 of the LOM is separated into an electrochemical (1E) step in which the ligand hole is generated and a chemical step (1C) in which the lattice oxygen is exchanged into the adsorbed intermediate. For both (**a**,**b**) lattice species are shown in red and electrolyte species are shown in blue. In the PDOS diagrams, the electrolyte species are shown to the left of the energy axis and the crystal PDOS are shown to the right. (**c**) Correlation of oxygen evolution activity with the vacancy parameter *δ*. The vacancy parameter is indicative of the underlying electronic structure where vacancies are generated when there is significant Co 3d and O 2p band overlap. (**d**) Correlation of oxygen evolution activity with the oxygen ion diffusion rate, indicating that increased surface exchange kinetics trend with increased OER activity. Error bars represent standard deviation of triplicate measurements.

The catalytic activity towards the OER was found to strongly correlate with the oxygen diffusion rate and the vacancy concentration, *δ*, presented in Fig. 6c,d. On the basis of these correlations, we hypothesize a new OER mechanism in Fig. 6a based on the exchange of lattice oxygen species that takes into account the role of surface oxygen vacancies and B–O bond covalency (lattice oxygen-mediated OER, LOM). In contrast to the general adsorbate evolution mechanism (AEM) which considers only the redox activity of the transition metal B-site, we find a better electronic explanation arises when the covalency of the M–O bond is considered, indicative of the overlap of the Co 3d and O 2p bands in the crystal, as first proposed by Matsumoto *et al*.^14^,^45^. As the oxidation state of Co is increased, the d orbitals of the Co ion have a greater overlap with the s, p orbitals of the O^2−^ ion, leading to the formation of π* and σ* bands, as described through Fig. 1 and in the partial density of states (PDOS) diagrams in Fig. 6a and refs 11, 13, 22, 43. When the overlap is great enough, ligand holes (oxygen vacancies) are formed and the metal 3d π* band can no longer be treated as isolated in energy from the oxygen O 2p π* band. At this point, the surface of the crystal and bound intermediates can be treated as a single energy surface, where the Fermi energy can be modulated through the hybridized Co 3d–O 2p π* band with applied electrical potential, opening up the possibility for lattice oxygen redox activity^46^. A recent *in situ* ambient pressure XPS study has confirmed the validity of this model in perovskites and other oxides^47^,^48^. In addition, oxygen redox activity has been observed in LSCO with high Sr^2+^ content in the regime of oxygen intercalation, which occurs approximately at the onset potentials of the OER in these materials^49^,^50^,^51^.

To test the validity of this lattice oxygen-mediated mechanism (LOM) and identify the rate-determining step, we modelled the reaction pathway using density functional theory^52^. Supplementary Fig. 7a shows that OH^−^_(aq)_ tends to electrochemically fill the surface O vacancies of LSCO under the operational electrode potential of OER, as described through reaction 3 and LOM 1 in Fig. 6a, leading to an *in situ* surface–layer stoichiometry close to that of stoichiometric bulk ABO_3_. Consequently, we begin by constructing the [001] BO_2_ terminated surfaces (Supplementary Fig. 8a) with ¼ ML OER intermediate adsorbates^52^ based on the 2 × 2 × 2 cubic stoichiometric bulk LSCO for the initial identification of the reactivity trend and reaction mechanism^53^. We subsequently investigated more realistic bulk phases with oxygen vacancies and various surface structures, which we find do not alter the preference of LOM over AEM; further details of these computations are provided in the Supplementary Methods.

**Figure 7:**
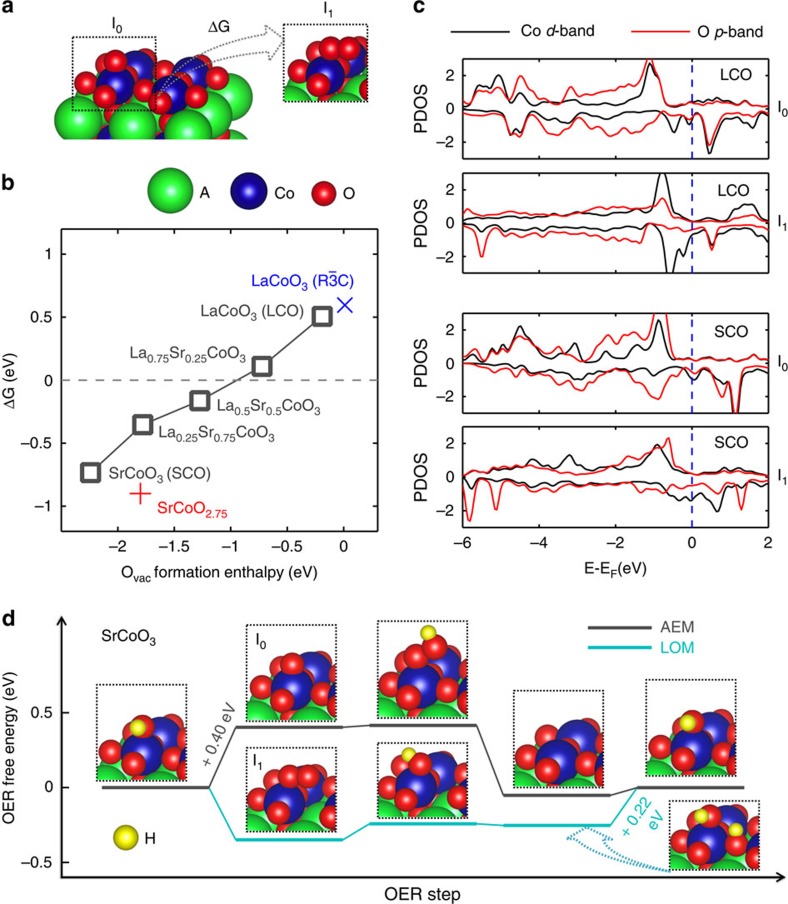
Density functional theory modelling of vacancy-mediated oxygen evolution on La_1−*x*_Sr_*x*_CoO_3−*δ*._ (**a**) Surface configurations of the intermediate after AEM Step 1 (I_0_) and the one after LOM Step 1 (I_1_). (**b**) The free energy change of I_1_ over I_0_ versus the O vacancy formation enthalpy in the bulk, for the cubic La_1−*x*_Sr_*x*_CoO_3−*δ*_ (black mark), where *x*=0, 0.25, 0.5, 0.75 and 1, with the rhombohedral LaCoO_3_ and optimized SrCoO_2.75_ phases; for *x*=0.25 and 0.75, the most energetic favourable vacancy site is selected; the O vacancy formation energy is calculated at the concentration of 1 per 2 × 2 × 2 unit cell with respect to H_2_O(g) and H_2_(g) at standard condition; using O_2_(g) as the reference will shift the O vacancy formation enthalpy around +2.5 eV larger. (**c**) The density of states of d-band for the active surface Co and the overall p-band for its ligand O, for LaCoO_3_ and SrCoO_3_ before and after the lattice oxygen exchange. (**d**) The OER free energy changes of LOM and AEM on SrCoO_3_ at the concentration of ¼ ML, with indicated intermediates structures and potential-determining steps.

Our results show that Step 1 differentiates the LOM, involving the intermediate with adsorbed –OO and lattice O vacancies (I_1_ in Figs 6a and 7a), from the AEM, involving the generally proposed adsorbed –O (I_0_ in Figs 6a and 7a). Therefore, the relative stabilities (free energy difference, ΔG) between these two isomeric intermediates are key to identifying if OER proceeds via the LOM or AEM for a given LSCO composition. This identification approach has been successfully used to demonstrate the preference of LOM on LaNiO_3_ (ref. 52). The computed values of ΔG are shown as a function of LSCO composition in Fig. 7b, which illustrates two key points. First, increasing *x* in La_1−*x*_Sr_*x*_CoO_3−*δ*_ reduces the O vacancy formation energy and therefore bulk stability. Second, ΔG decreases with the decreased bulk stability, becoming negative between 0.25<*x*<0.5. Therefore, OER on perovskites with low stability such as La_0.5_Sr_0.5_CoO_3−*δ*_, La_0.25_Sr_0.75_CoO_3−*δ*_ and SrCoO_3−*δ*_ is predicted to occur via the LOM, whereas LaCoO_3_ and La_0.75_Sr_0.25_CoO_3−*δ*_are expected to follow the AEM.

The transition from the AEM to the LOM is related to the ineffectiveness of the surface Co as electron donors. The double bond of the adsorbed O formed in reaction 1 of the AEM significantly increases the oxidation state of surface Co to >3+. In the LOM step 1, the transfer of a surface O to form a surface O vacancy and the single-bonded –OO adsorbate decreases the nominal valence charge on the Co to 3+. Thus, the LOM pathway has higher stability than the AEM pathway, particularly for those LSCO with large *x*. The relative stability of I_1_ to I_0_ is also apparent in the projected density of states of the d-band for the active surface Co and the overall p-band for its ligand O (Fig. 7c). The overlap of the peaks in these two bands indicates the orbital hybridization and Co–O binding. For the AEM intermediate on LaCoO_3_ (I_0_), the strong overlap of peaks in the spin-up (down) bands centred around −1 eV (0.5 eV) indicates the strong Co–O covalent bonding state. These overlaps, however, are significantly weakened for I_1_, consistent with the stability. The reverse is true for the LSCO with low stability. Compared with I_0_ for SrCoO_3_, I_1_ preserves a significant overlap of spin-up state around −1 eV, but has negligible overlap of the unoccupied spin-down states, which are anti-bonding in character, indicating the greater stability of I_1_.

To understand the phase and stoichiometry effects on the relative stability of I_1_ to I_0_, we perform the analogous calculations on the rhombohedral LaCoO_3_ and the nonstoichiometric SrCoO_2.7_ phases. The rhombohedral LaCoO_3_ phase is modelled by optimizing an initial 2 × 2 × 2 orthorhombic cell with octahedral rotation; the optimized structure exhibits a Co–O–Co angle of 162° and a Co–O distance of 1.96 Å, consistent with experimental measurements. The SrCoO_2.7_ phase is approximated as SrCoO_2.75_, which can be modelled by relaxing the cubic 2 × 2 × 2 SrCoO_3_ structure with two oxygen vacancies. By comprehensively searching the vacancy ordering, we identify the most stable configuration as the presence of the two vacancies surrounding one Co, which therefore leads to the formation of a tetrahedral CoO_4_ linked to two tetragonal pyramidal CoO_5_ units (Supplementary Fig. 8b). The lattice constant of this optimized SrCoO_2.75_ is within 1.1% difference from that of the derived pseudocubic SrCoO_2.7_ (Supplementary Table 1). This configuration is further validated by introducing two more vacancies to form SrCoO_2.5_ in the same way, so as to maximize the number of tetrahedral CoO_4_. The relaxed SrCoO_2.5_ shows alternating octahedral (CoO_2_) and tetrahedral zigzag-like (CoO) layers with respect to the (001) direction of the reference cubic phase (Supplementary Fig. 8b), in full agreement with experimental observations. The SrCoO_2.75_ slab is subsequently constructed by exposing the (CoO_2-*δ*_) layer (Supplementary Fig. 8c), but with added oxygen anions to attain the correct stoichiometry (Supplementary Fig. 8d) to simulate the intercalation phenomenon as described by Supplementary Fig. 7a and LOM Step 3. As Fig. 7b shows, the octahedral rotation stabilizes the rhombohedral LaCoO_3_, leading to a slight increase in the oxygen vacancy formation energy and ΔG. In the case of SrCoO_2.75_, the existing oxygen deficiency increases the oxygen vacancy formation energy by 0.33 eV, while slightly stabilizing I_1_ relative to I_0_, compared with SrCoO_3_. The lattice constant of the predicted SrCoO_2.75_ is 0.7% larger than that of SrCoO_3_, leading to the slightly weaker adsorption strength and lower stability of I_0_ (ref. 54). However, the small magnitude of this change indicates the similar reactivity of SrCoO_3_ to that of the intercalated SrCoO_2.7_ surface under OER conditions. From the above analysis, we conclude that neither the phase nor the non-stoichiometry alters the qualitative stability of I_1_ to I_0_, although it leads to a horizontal shift in the overall trend of bulk vacancy formation to higher energetic cost.

We also compute the free energy of electrochemical OER on SrCoO_3_ to demonstrate the switch in the reaction mechanism due to the relative change in I_1_-to-I_0_ stability on SrCoO_2.7_. In accordance with the procedure in ref. 53 the free energy of each reaction step is determined by *ΔG*_*R*_=*E+ΔZPE−TΔS−eU*_RHE_ at *U*_RHE_=+1.23 V, where *ΔE* is the DFT-computed enthalpy change for ¼ ML of intermediates relative to H_2_O and H_2_ molecules (Supplementary Table 3) and *ΔZPE*—*TΔS* gives the corrections for zero-point energy and entropy of both adsorbates and H_2_(g) and H_2_O (l) under OER conditions (Supplementary Table 4) (refs 53, 54). The largest free energy is the estimated overpotential, *η*. As *ΔG*_*R*_ is independent of the initial OER intermediate considered, we—in practice—start from the stoichiometric hydroxylated surface (the surface before LOM 1). Figure 7d shows that the first step (−OH to I_0_) of AEM is the potential-determining step, with *η*=0.4 V. However, it becomes remarkably energetically favourable to follow LOM 1, forming the superoxide-like –OO (*V*_O_) adsorbates (I_1_) with an O-to-O bond length of 1.28 Å. Therefore, LOM is the relevant mechanism for SrCoO_2.7_. Once I_1_ forms, it requires small energetically uphill and downhill reactions, respectively, to evolve back to –OH (*V*_O_) and electrochemically fill the vacancy by OH^−^ (aq) in Step 2 and 3 of the LOM. This electrochemical surface hydroxylation during Step 3 occurs concomitantly with an electron transfer to leave the surface in a neutral state. The subsequent step of electrochemical deprotonation is identified as the potential-determining step, similar to the results for LaNiO_3_ (ref. 52). Further, the computed overpotential of 0.22 V is fully consistent with experiments.

We note that consideration of modified surface configurations, which may occur under operating conditions, could lead to different values of ΔG. For example, full surface hydroxylation can further decrease the value of ΔG due to oxidation of the surface Co, making the LOM more favourable, while moderate protonation of surface oxygen can increase ΔG by donating electrons to the surface. In addition, the O-to-O overbinding effects in the superoxide formation (I_1_) by RPBE can increase ΔG by <0.3 eV (ref. 55), while the use of GGA+*U*_eff_ can lead to the weaker adsorption strength of I_0_, decreasing ΔG by >0.3 eV (ref. 56). Nevertheless, the behaviour of the model surfaces expected to be qualitatively correct for these systems, and also independent of exchange correlation functional, as demonstrated for LaNiO_3_.

Interestingly, significant oxygen deficiencies of the LSCO series begin to appear at *x*=0.4, matching well with the predicted transition from the AEM to LOM at 0.25<*x*<0.50. These oxygen deficiencies reveal the saturated charge states of Co, which become unable to donate enough electrons to attain oxygen stoichiometry as described through Fig. 1. The bulk oxygen deficiency is consequently indicative of the LOM, since the double bonded –O (AEM) induces a higher oxidation state of the surface Co than that in the bulk. The transition is further demonstrated by the experimental observation that the current density at *U*_RHE_=+1.63 V increases on a very different scale with increasing *δ* when *x*>0.4 from that when *x*<0.4. Our work thus provides a strong theoretical framework, consistent with experiments, to describe the transition of the OER mechanism as a function of bulk stability. Further discussion about the applicability of this mechanism to other metal oxide catalysts is included in Supplementary Fig. 9 and in the Supplementary Discussion.

## Discussion

We have demonstrated that oxygen vacancy defects are a crucial parameter in improving the electrocatalysis of oxygen on metal oxide surfaces, whereby they may control the physical parameters of ionic diffusion rates and reflect the underlying electronic structure of the catalyst. The vacancy-mediated mechanism proposed offers insight into the design of highly active OER catalysts, and allows for the rationalization of the electrolysis of water using surface chemistry parameters, as described through the modulation of the Fermi energy through transition metal 3d and oxygen 2p partial density of states at the surface. As such, the role of oxygen vacancy defects cannot be ignored, and should be a critical component in the benchmarking of metal oxide oxygen electrocatalysts and the advancement of the mechanistic theory behind the OER.

## Methods

### General

All chemicals were used as received. Anhydrous ethanol and 5 wt% Nafion solution in lower alcohols were purchased from Sigma-Aldrich. Lanthanum (III) nitrate hexahydrate (99.999%), strontium (II) nitrate hexahydrate (99.9%), cobalt (II) nitrate hexahydrate (99.9%), tetrapropylammonium bromide (98%), tetramethylammonium hydroxide (TMAH) pentahydrate (99%), 2-propanol, potassium hydroxide, potassium iodide (≥99%), sodium thiosulfate (0.1 N), potassium iodate (0.1 N) and hydrochloric acid were obtained from Fisher Scientific. Absolute ethanol (200 proof) was obtained from Aaper alcohol. The commercial IrO_2_ sample was obtained from Strem Chemicals. Oxygen (99.999%) and argon (99.999%) gases were obtained from Praxair. VC was obtained from Cabot Corporation and the NC was prepared as reported elsewhere^57^.

### Synthesis of La_1−*x*_Sr_*x*_CoO_3−*δ*,_ 0≤*x*≤0.8

La_1−*x*_Sr_*x*_CoO_3−*δ*_ was synthesized following our previously reported reverse-phase hydrolysis approach^16^,^17^,^27^. Mixed metal hydroxides were prepared by reverse-phase hydrolysis of La, Sr and Co nitrates in the presence of an equimolar amount of tetraproprylammonium bromide (TPAB) dissolved in 1 wt% TMAH. An ∼10 mM solution of mixed metal nitrates of the appropriate stoichiometry was added dropwise at ∼1–2 ml min^−1^ to 200 ml of the 1 wt% TMAH solution containing TPAB. The resulting precipitated mixed metal hydroxide nanoparticles were collected by centrifugation and washed with deionized water, followed by re-suspension in deionized water through probe sonication. The solution was frozen as a thin film on a rotating steel drum at cryogenic temperatures (−79 °C), and then lyophilized at −10 °C at a fixed pressure of ∼50 mTorr for 20 h. The lyophilized powder was calcined in a tube furnace under dehumidified air at a flow rate of 150 ml min^−1^ for 5 h at 950 °C. The resulting perovskites are then washed with ethanol followed by water and allowed to dry in an oven at 80 °C overnight.

### Synthesis of SrCoO_2.7_

Synthesis of SrCoO_2.7_ followed a similar procedure to the one used above, but used a slower addition rate of metal nitrate solution to TMAH/TPAB of <0.5 ml min^−1^. In addition, the hydrolysis reaction was allowed to proceed for 5 days before collection by centrifugation. Finally, the flow rate of dehumidified air during calcination was adjusted to 20 ml min^−1^.

### Materials characterization

Bulk crystal structures were determined through wide-angle X-ray diffraction (Rigaku Spider, Cu Kα radiation, *λ*=1.5418 Å) and analysed with JANA2006 software^58^. The TEM samples were prepared by crushing the crystals in a mortar in ethanol and depositing drops of suspension onto holey carbon grid. Electron diffraction patterns, TEM images, HAADF-STEM images, ABF-STEM images and energy dispersive X-ray spectra were obtained with an aberration-corrected Titan G^3^ electron microscope operated at 200 kV using a convergence semi-angle of 21.6 mrad. The HAADF and ABF inner collection semi-angles were 70 mrad and 10 mrad, respectively. Iodometric titrations were performed according to the referenced procedure^19^. In short, 3 ml of de-oxygenated 2 M KI solution was added to a flask containing 15–20 mg of perovskite under an Ar atmosphere and allowed to disperse for three minutes. After a few minutes 25 ml of 1 M HCl is added and the perovskite is allowed to dissolve. This solution is then titrated to a faint golden colour with a solution of ∼40 μM solution of Na_2_S_2_O_3_ that has been pre-standardized with 0.1 N KIO_3_. Starch indicator is then added and the solution is titrated until clear, marking the end point. BET surface area measurements were performed through nitrogen sorption on a Quantachrome Instruments NOVA 2000 high-speed surface area BET analyser at a temperature of 77 K, using 7 points from the linear region of the adsorption isotherm to determine the surface area.

### Electrode preparation

All La_1−*x*_Sr_*x*_CoO_3−*δ*_ nanopowders and the commercial IrO_2_ sample were loaded onto carbon through ball milling with a Wig-L-Bug ball mill. For rotating disk electrode (RDE) and for the RRDE measurements the LSCO nanopowders were loaded at a mass loading of ∼30 wt% onto NC. For the galvanostatic stability tests, LSCO nanopowders and IrO_2_ were also loaded onto VC (XC-72, Cabot Corporation) at a mass loading of ∼30 wt%. The LSCO/carbon mixtures were dispersed in ethanol containing 0.05 wt% Na-substituted Nafion at a ratio of 1 mg ml^−1^ and sonicated for 45 min. This solution was spuncast onto a glassy carbon RDE (0.196 cm^2^_geom_, Pine Instruments) and for the RRDE (Glassy Carbon Disk: 0.2472, cm^2^_geom_; Pt ring: 0.1859, cm^2^_geom_, Pine Instruments) at a total mass loading of 51.0 μg cm^−2^_geom,disk_ (LSCO loading: 15.3 μg cm^−2^_geom_). The synthesis of the NC is described elsewhere^57^. For the oxygen intercalation cyclic voltammetry studies the LSCO nanopowders were loaded at a mass loading of 85 wt% on VC (Cabot Corporation). The LSCO/carbon mixtures were dispersed in ethanol containing 0.1 wt% Na-substituted Nafion at a ratio of 2 mg ml^−1^ and sonicated for 45 min. This solution was spun cast onto the glassy carbon RDE at a total mass loading of 102.0 μg cm^−2^_geom_ (LSCO loading 86.7 μg cm^−2^_geom_). The electrodes were cleaned before spin casting by sonication in a 1:1 deionized water:ethanol solution. The electrodes were then polished using 50 nm alumina powder, sonicated in a fresh deionized water:ethanol solution and dried under a scintillation vial in ambient air.

### Electrochemical testing

Electrochemical testing was performed on a CH Instruments CHI832a potentiostat or a Metrohm Autolab PGSTAT302N potentiostat, both equipped with high-speed rotators from Pine Instruments. For the OER studies, the testing was done at room temperature in O_2_ saturated 0.1 M KOH (measured pH≈12.6). The current interrupt and positive-feedback methods were used to determine electrolyte resistance (50 Ω) and all data was *iR* compensated after testing. Each measurement was performed in a standard three-electrode cell using a Hg/HgO (1 M KOH) reference electrode, a Pt wire counter electrode, and a film of catalyst ink on the glassy carbon working electrode. All OER testing was performed on a new electrode that had not undergone previous testing. Cyclic voltammetry was performed from +0.9 to +1.943 V at 10 mV s^−1^ with a rotation rate of 1,600 r.p.m. To compensate for capacitive effects, the currents were averaged for the forward and backwards scans (Supplementary Fig. 10) The current at +1.63 V was selected from the polarization curves to compare the OER activities. For the rotating-ring-disk studies, the same parameters were used for the disk and the Pt ring electrode was held at a constant potential of +0.4 V versus RHE for the reduction of O_2_ to OH^−^. The Pt ring of the RRDE was electrochemically cleaned before testing by cyclic voltammetry on only the polished electrode in 0.1 M KOH through the hydrogen reduction potential regime at 5 mV s^−1^ for 20 cycles. The collection efficiency of the RRDE was measured as *N*=0.37 through calibration in 0.3 mM Ferrocene-methanol in 0.1 M KCl electrolyte (Supplementary Fig. 11). Stability tests were performed galvanostatically at a current density of 10 A g^−1^_ox_ and a rotation rate of 1,600 r.p.m. for 24 h for SrCoO_2.7_ and IrO_2_ supported on either NC or on VC. A cutoff potential of +1.75 V versus RHE was used to stop the test to preserve the integrity of the glassy carbon electrode supports. All potential are reported versus the RHE, which was measured as E_RHE_=E_Hg/HgO_+0.8456 V through the reduction of hydrogen in 1 atm H_2_ saturated 0.1 M KOH (Supplementary Fig. 12).

### Oxygen intercalation and diffusion rate measurements

The reversible intercalation of oxygen into LSCO was measured using cyclic voltammetry in an Ar saturated 1 M KOH electrolyte at 20 mV s^−1^ in a standard 3-electrode cell, using a Hg/HgO (1 M KOH) reference electrode, a Pt wire counter electrode, and a working electrode of a thin film of LSCO/VC on a glassy carbon electrode as described above. The electrodes were stationary during testing and cycled twice. The data shown is from the second cycle. Following this, the diffusion rates of oxygen in the crystal were measured based on an adaptation of the procedure given in refs 39, 40, 41. In short, following the cyclic voltammetry oxygen intercalation measurements, the E_1/2_ of the intercalation redox peaks was determined as the potential half way between the peak currents for intercalation and de-intercalation. The same electrodes were tested chronoamperometrically by applying a potential 50 mV more anodic of the *E*_1/2_. The electrodes were rotated at 1,600 r.p.m. to get rid of electrolyte based mass-transfer effects, and the current was measured as a function of time for 4 h. The current was plotted versus *t*^−1/2^ and the linear section of the curve was fit to find the intercept with the *t*^−1/2^ axis. Using a bounded 3-dimensional solid-state diffusion model, this intersect is indicative of the diffusion rate of oxygen according to the relation 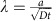, where, *λ* is a shape factor for the particles (in this case *λ*=2 for rounded paralelipipeds), *a* is the radius of the particle (in this case 150 nm was used for all LSCO samples), *t*^−1/2^ is determined from the intersection with the *t*^−1/2^ axis, and *D* is the diffusion rate of oxygen ions in the crystal measured at room temperature.

### Density function theory calculations and surface models

DFT calculations^55^,^59^,^60^ are performed using VASP with PAW pseudopotentials and the RPBE-GGA functional. More details are provided in the Supplementary Methods.

## Additional information

**How to cite this article:** Mefford, J. T. *et al*. Water electrolysis on La_1−*x*_Sr_*x*_CoO_3−*δ*_ perovskite electrocatalysts. *Nat. Commun.* 7:11053 doi: 10.1038/ncomms11053 (2016).

## Supplementary Material

Supplementary Information Supplementary Figures 1-12, Supplementary Tables 1-5, Supplementary Discussion, Supplementary Methods and Supplementary References

## Figures and Tables

**Table 1:** Oxygen vacancy concentration, *δ*, and cobalt oxidation state, *y*.

x in La_1−*x*_Sr_*x*_CoO_3−*δ*_	*δ*	*y*
0	−0.01±0.01	3.01±0.01
0.2	0.01±0.01	3.18±0.02
0.4	0.05±0.04	3.30±0.08
0.6	0.09±0.01	3.43±0.01
0.8	0.16±0.01	3.48±0.02
1.0	0.30±0.03	3.40±0.06

Error is based on the s.d. of triplicate measurements.
