# Supplementary material for: Water electrolysis on La1−xSrxCoO3−δ perovskite electrocatalysts
Source: Nat Commun. 2016 Mar 23;7:11053. doi: 10.1038/ncomms11053 (PMC4814573; doi:10.1038/ncomms11053)
Supplement: Supplementary Information — Supplementary Figures 1-12, Supplementary Tables 1-5, Supplementary Discussion, Supplementary Methods and Supplementary References [file ncomms11053-s1.pdf]

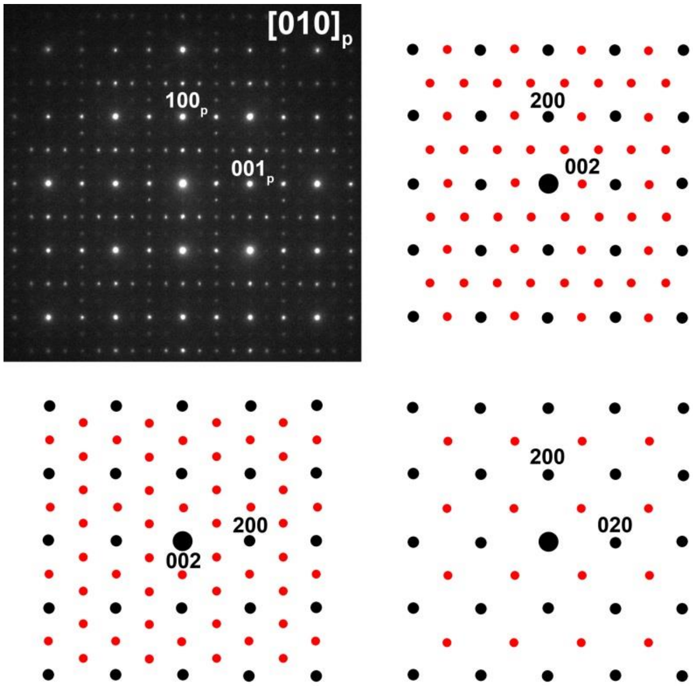

Supplementary Figure 1: Decomposition of the $[010]_p$ SAED pattern of SCO into contribution of the twinned domains. Three twinned superstructure variants contributing to the experimental pattern are shown schematically (the perovskite subcell reflections and reflections due to *I*-centered tetragonal $2a_p \times 2a_p \times 4a_p$ supercell are shown as black and red dots, respectively).

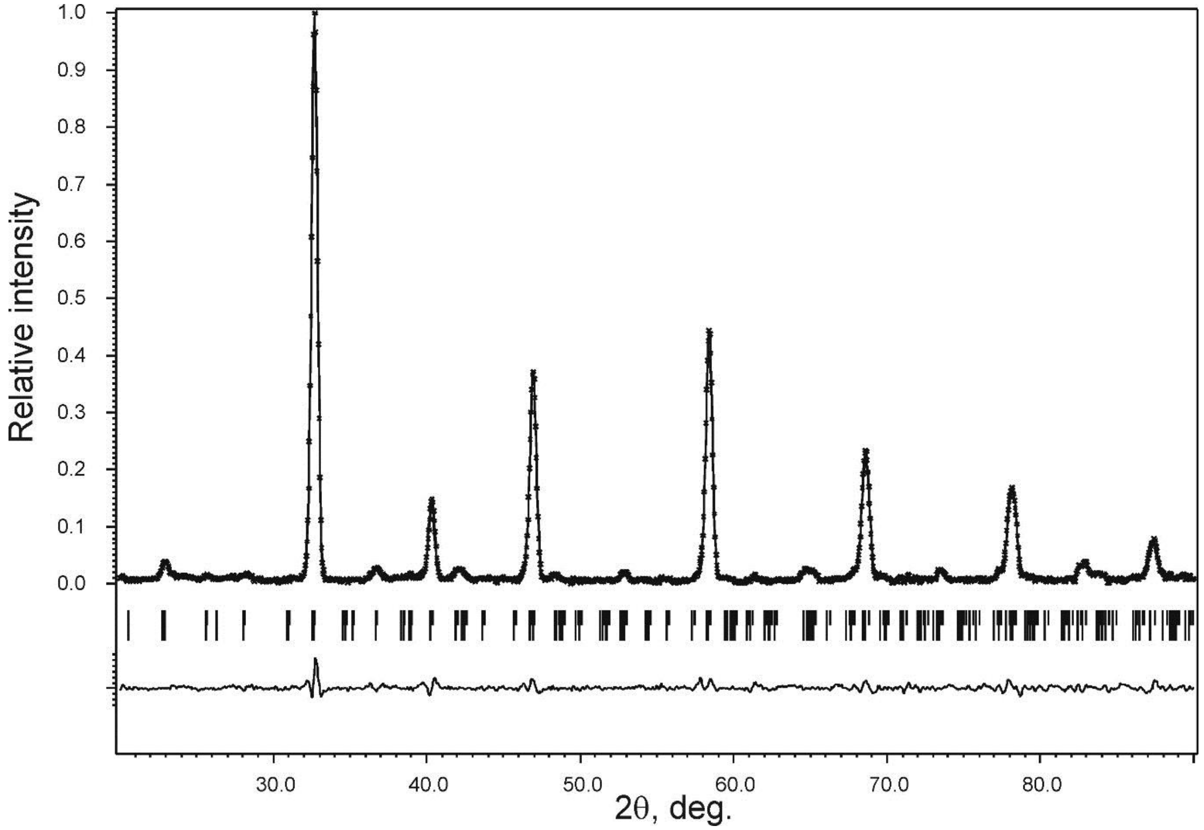

Supplementary Figure 2: LeBail fit of the PRXD pattern of SCO. The bars mark the reflection positions according to the body-centered tetragonal $2a_p \times 2a_p \times 4a_p$ supercell.

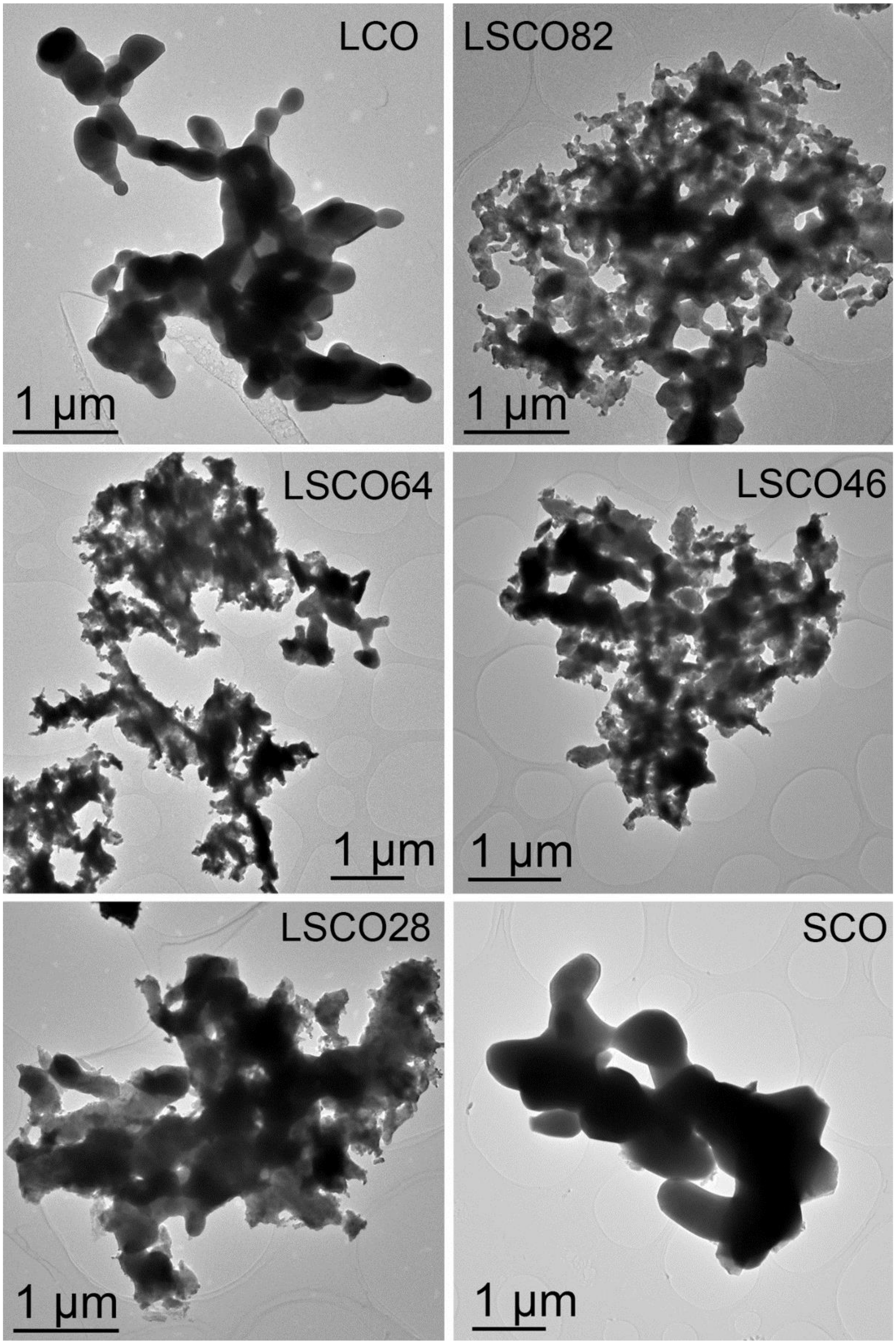

Supplementary Figure 3: Bright field TEM images of the LCO, LSCO and SCO samples.

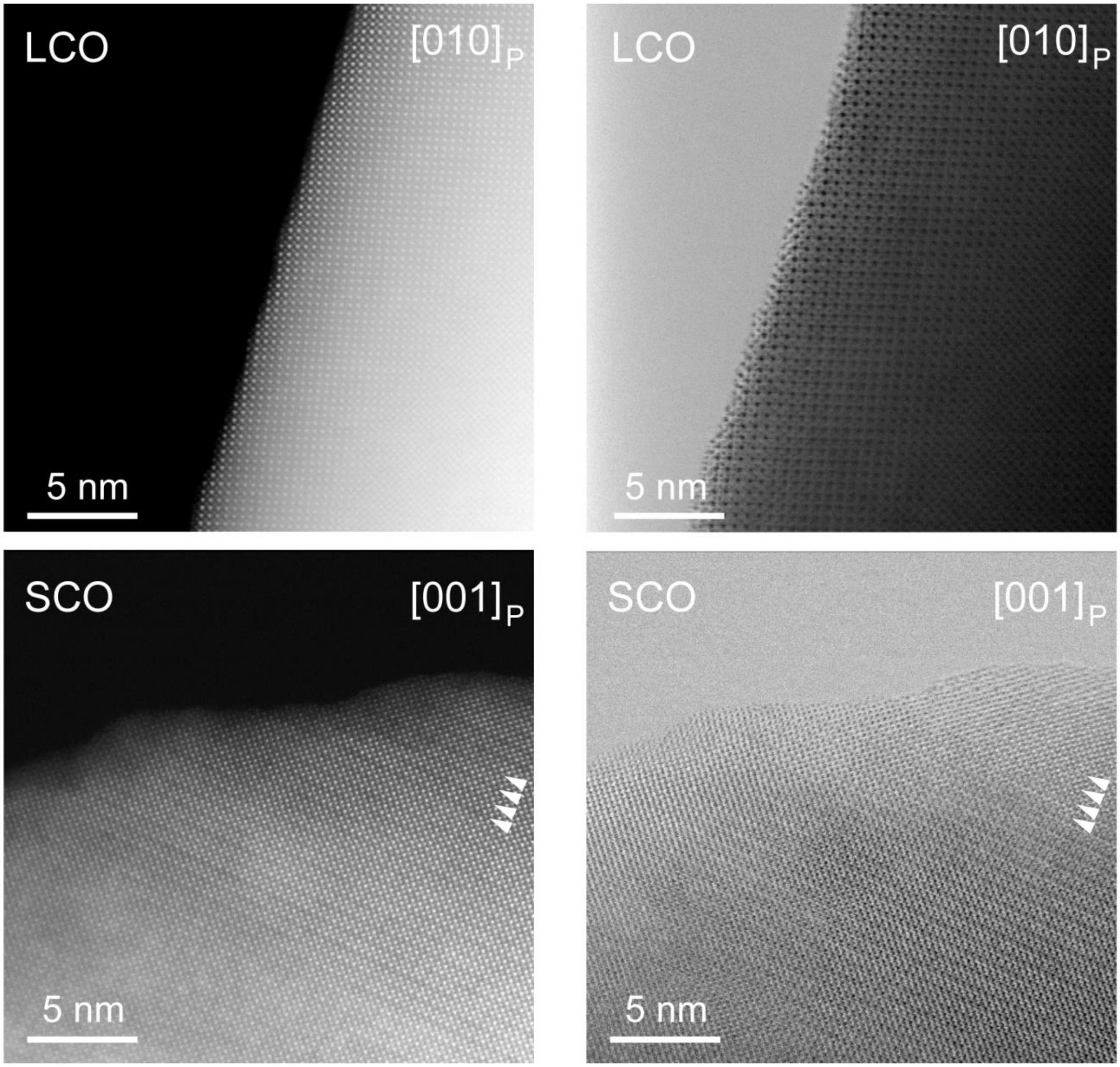

Supplementary Figure 4: HAADF-STEM (left) and ABF-STEM (right) images of the LCO and SCO samples. Note the absence of the amorphous surface layer for LCO as well as for SCO. In addition, the arrangement of alternating (CoO_2) perovskite layers and ($CoO_{2-\delta}$) anion-deficient layers extends to the surface of SCO (marked with arrowheads), indicating the presence of surface oxygen vacancies.

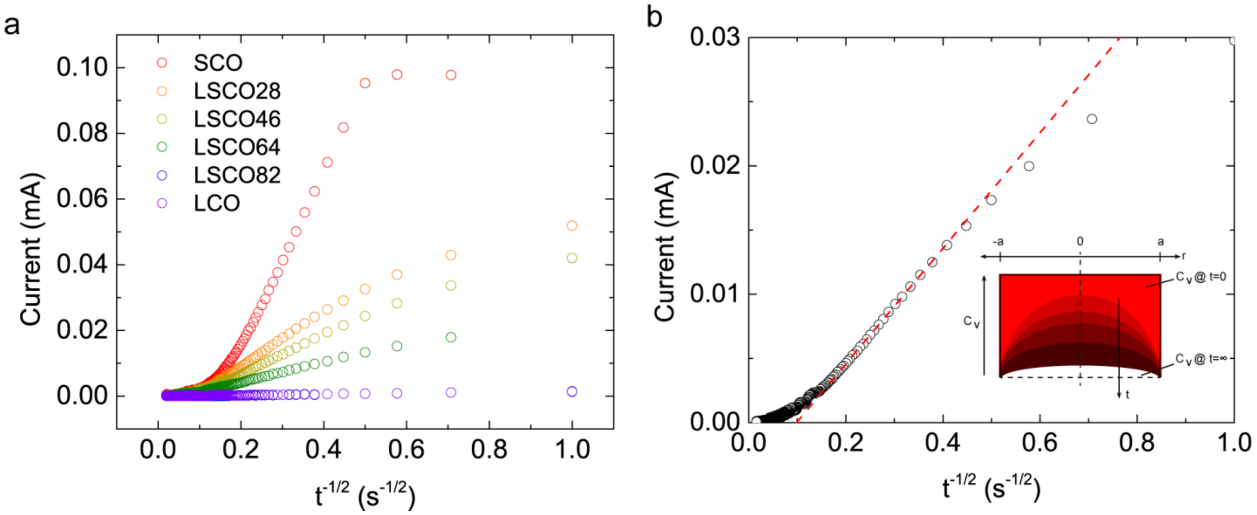

Supplementary Figure 5: Chronoamperometric oxygen diffusion rate measurements. (**a**) Current vs. $t^{-1/2}$ for $La_{1-x}Sr_xCoO_{3-\delta}$ ($0 \le x \le 1$). (**b**) Isolated current vs. $t^{-1/2}$ for $La_{0.4}Sr_{0.6}CoO_{3-\delta}$. The diffusion rate can be measured from the intersection of the linear portion of the current vs. $t^{-1/2}$ with the $t^{-1/2}$ axis. The theory is based on a bounded 3-dimensional diffusion model, with a diagram of the model shown in the inset of (**b**) where the intersection of i vs. $t^{-1/2}$ at i=0 corresponds to $\lambda=a/\sqrt{Dt}$, where λ is a dimensionless shape factor, a is the radius of the particle, D is the diffusion rate, and $t^{-1/2}$.[1–3] Given a, and choosing an appropriate shape factor, λ, the diffusion rate can thus be easily measured. For a full derivation of the model, see Supplementary References 3-5. In this case, λ was chosen as 2, which is representative of a rounded paralelipid, halfway between the values for a sphere (λ=1.77) and a cube (λ=2.26). The value of a=150 nm was used for all materials based on our previous work, and the relation of surface area to particle size for a sphere: $SA=6/2a\rho$, where ρ is the density of LSCO (i.e. ρ=7.235 g cm^{-3} for $LaCoO_3$).[4] Further, it has been shown that particle size distribution does not affect the measured diffusion rate significantly.[3]

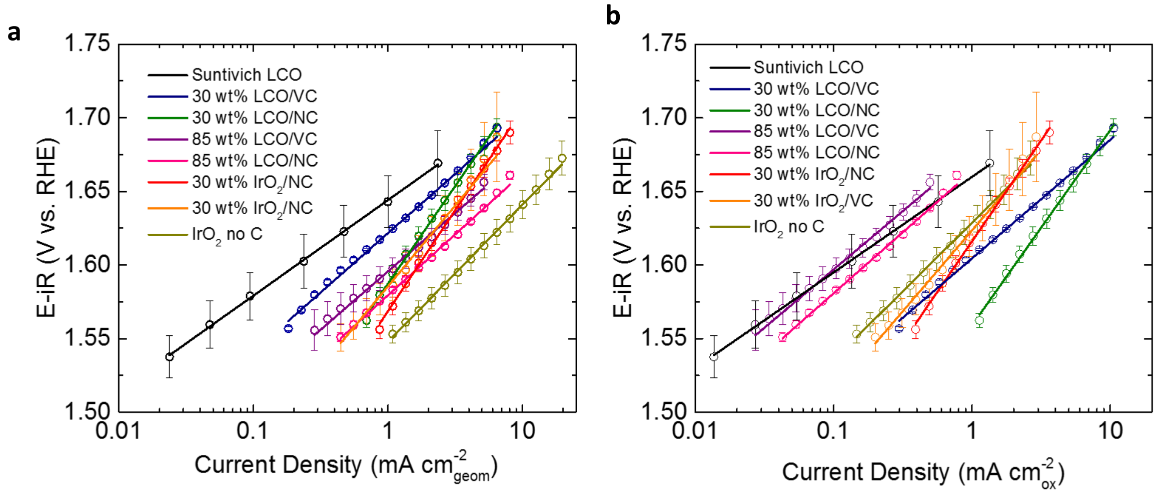

Supplementary Figure 6: (**a**) OER Tafel plot with geometric normalized current densities. (**b**) OER Tafel plot with BET surface area normalized current densities. Suntivich LCO refers to data for $LaCoO_3$ extrapolated from Supplementary Reference 8.[8]

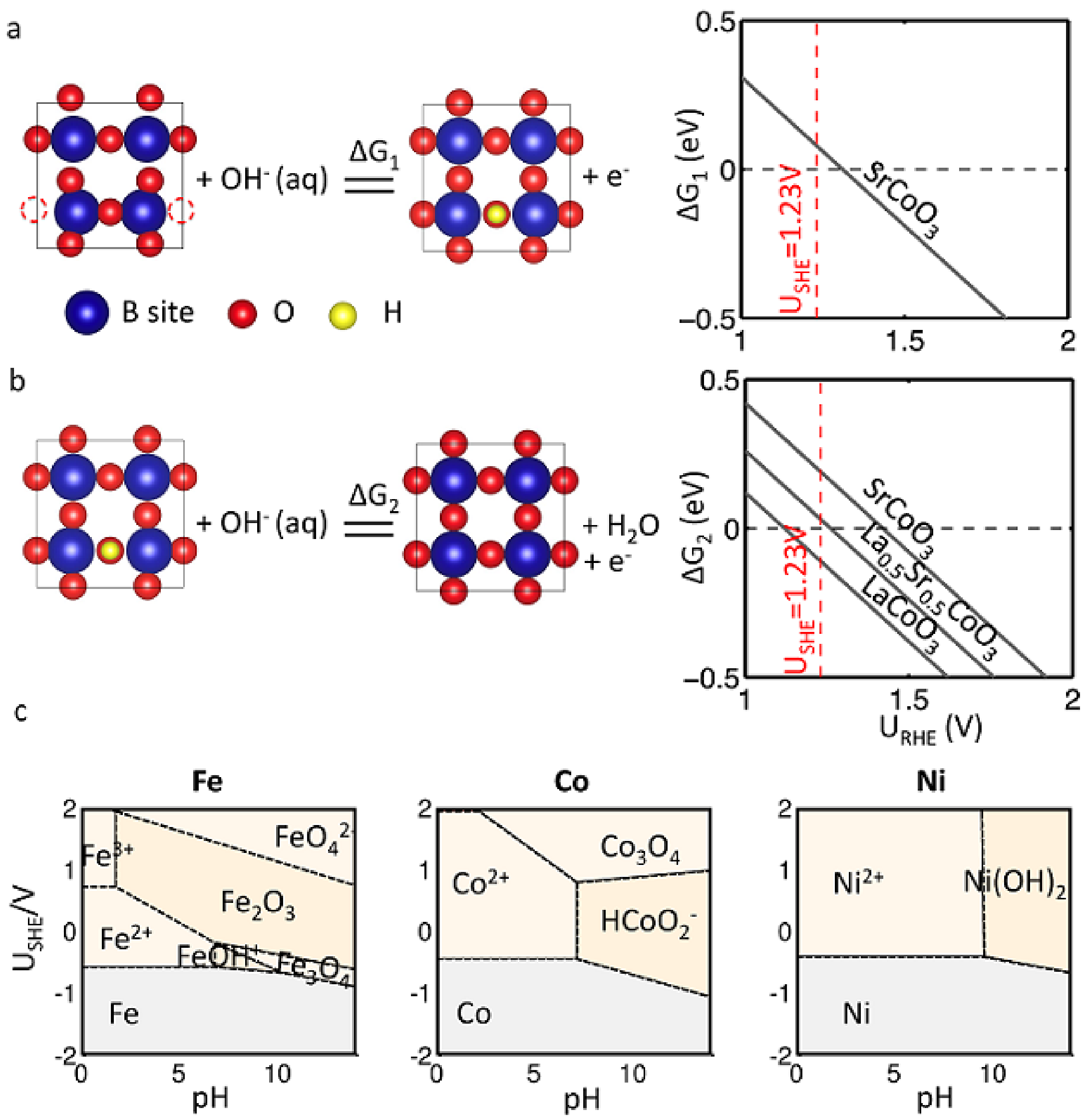

Supplementary Figure 7: (**a**) The reaction of the surface layer with O vacancy and $OH^-_{(aq)}$ to form the lattice hydroxides. (**b**) The reaction of the surface hydroxides and $OH^-_{(aq)}$ to form the clean surface. (**c**) Computed Pourbaix diagrams of Fe, Co and Ni in aqueous environment.[5] The reaction formation energy, ΔG, is equal to $\Delta E+\Delta ZPE-T\Delta S-U_{RHE}$, where ΔE is the formation enthalpy; ΔZPE is the difference of zero point energies; ΔS is the formation entropy, and U_{RHE} is the reversible hydrogen electrode potential. Details can be found in Supplementary Reference 6.[6]

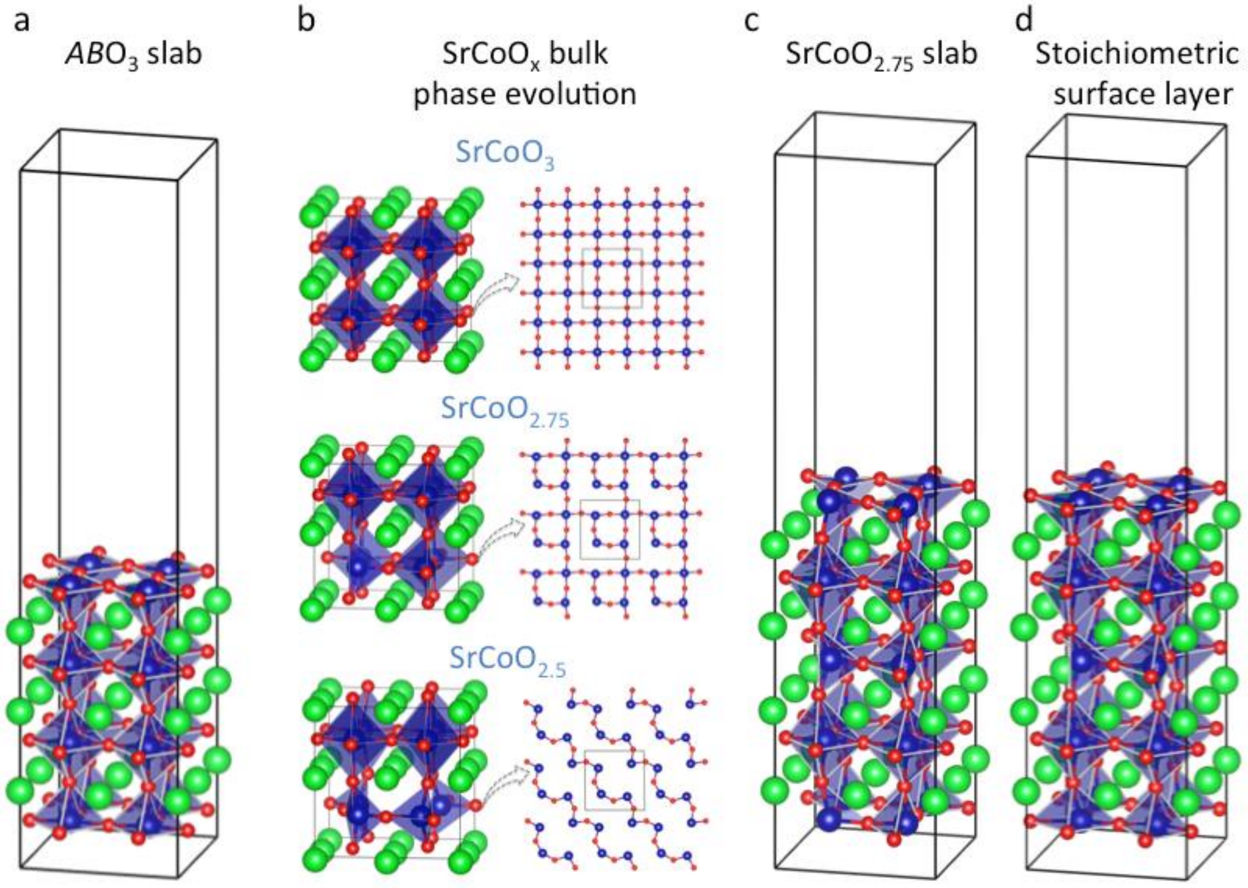

Supplementary Figure 8: (**a**) The slab model for the stoichiometric cubic LSCO. (**b**) The identification of $SrCoO_x$ phase evolution with respect to oxygen deficiency, with the representations of the bulk configuration (left panel) and the $CoO_{2-\delta}$ layer (right panel). The most stable $SrCoO_{2.75}$ and $SrCoO_{2.5}$ are comprehensively searched by inducing oxygen vacancies in $SrCoO_3$ and performing global relaxations, as mentioned in the manuscript (**c**) The slab model for $SrCoO_{2.75}$ with the exposed $CoO_{2-\delta}$ surface layer. (**d**) The slab model for $SrCoO_{2.75}$ with the intercalated and thus stoichiometric CoO_2 surface layer.

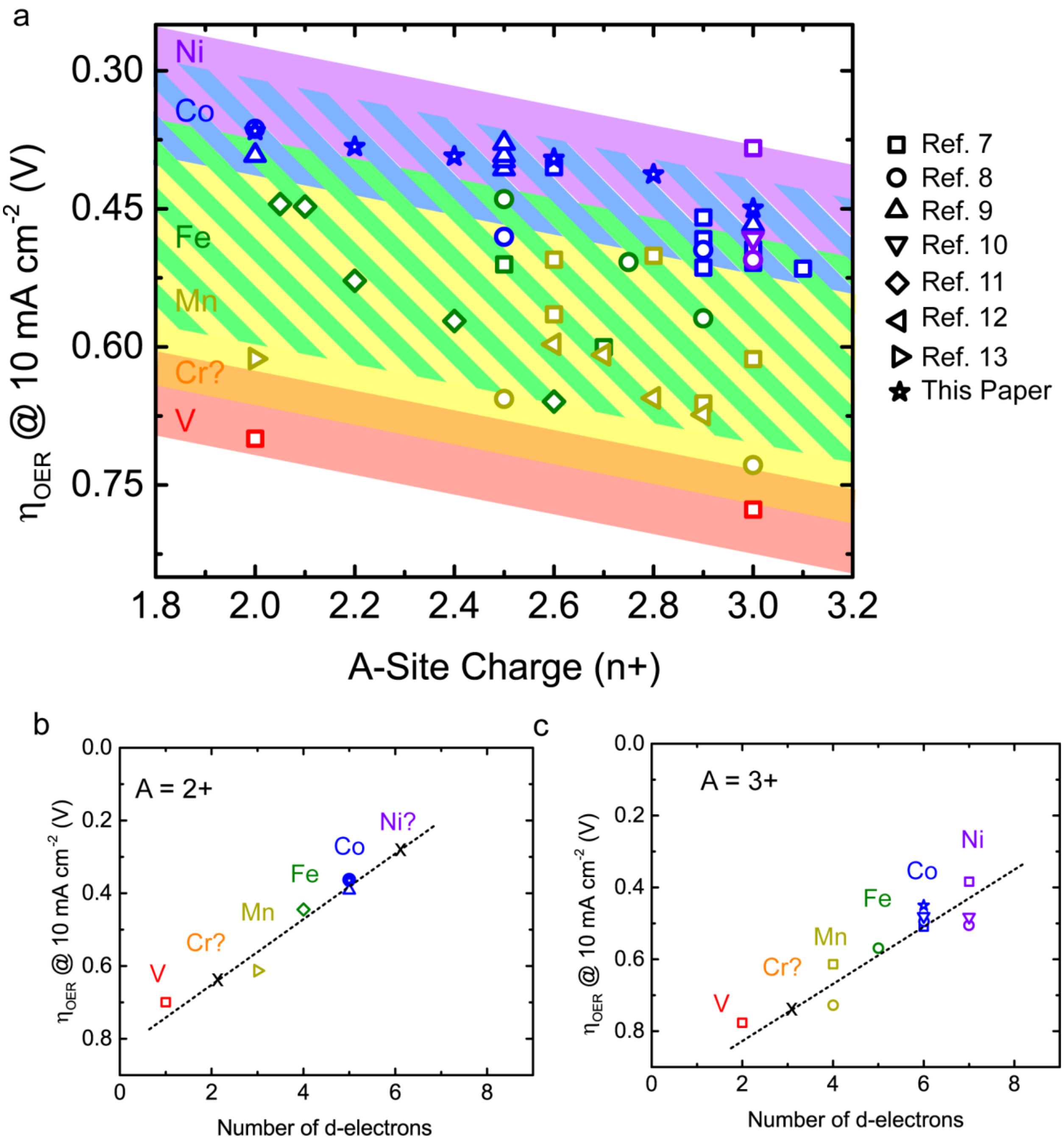

Supplementary Figure 9: Trends in OER activity with A-site charge and d-electron number. (**a**) The overpotential required to produce 10 mA cm^{-2} of current for the OER versus A-site charge.[7–13] Because many of the references did not measure the oxygen vacancy parameter, δ, the combined charge of A-site ions is used as a proxy for the vacancy concentration, with an increase in δ expected as the A-site charge is reduced. As can be seen, the overpotential is reduced as the A-site charge is reduced,

regardless of the transition metal element in the B-site of ABO_3. (**b**) Trends in overpotential for A-site charge = 2+ versus d-electron number. The covalency of the M-O bond is expected to increase with the electronegativity of the transition metal, which also increases with d-electron number.[14] (**c**) Trends in overpotential for A-site charge = 3+ versus d-electron number. In (**b,c**) materials with inconclusive data or those that which have not been synthesized in the literature, such as $A^{2+}NiO_{3-\delta}$ are marked with an **x**. The value of 10 mA cm^{-2} was chosen as the benchmark current density for solar to fuel generation from Supplementary Reference 15.[15]

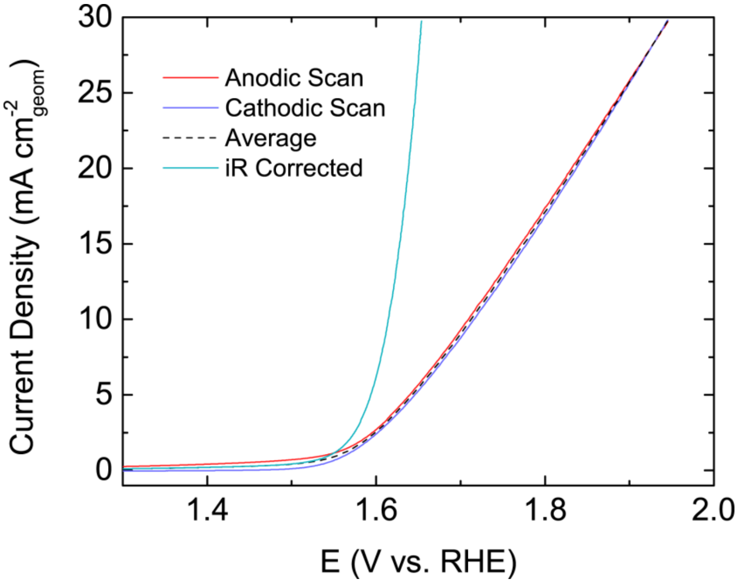

Supplementary Figure 10: Capacitance and iR correction of OER cyclic voltammograms. OER polarization curves for $SrCoO_{2.7}$ in O_2 saturated 0.1M KOH at 1600 rpm and a scan rate of 10 mV s^{-1}. The anodic and cathodic scans are shown prior to correction, as well as the capacitance corrected curve and the iR shifted curve. The low degree of hysteresis on the back scan is indicative that possible electrochemical or chemical side reactions contribute very little to the observed current.

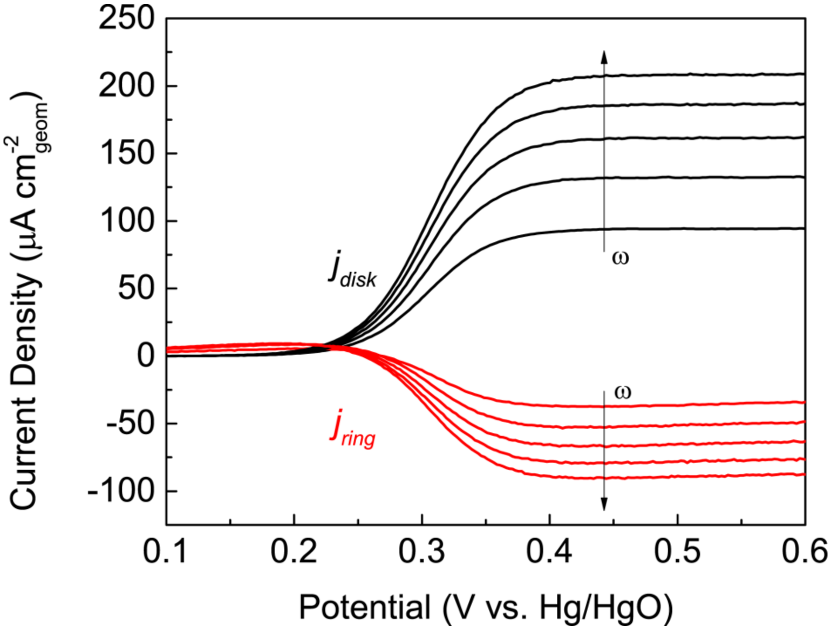

Supplementary Figure 11: Calibration of RRDE with Ferrocene-methanol. Linear sweep voltammetry was performed using a rotating ring-disk electrode (RRDE) with a glass carbon disk and a Pt ring in 0.3 mM ferrocene-methanol in 0.1M KCl. The disk potential was scanned from 0.1 to 0.6 V vs. Hg/HgO (1M KOH) while the ring was held at 0.1 V vs. Hg/HgO (1M KOH). Rotation rates of ω = 400, 800, 1200, 1600, and 2000 rpm were used. The collection efficiency was measured as N=0.37.

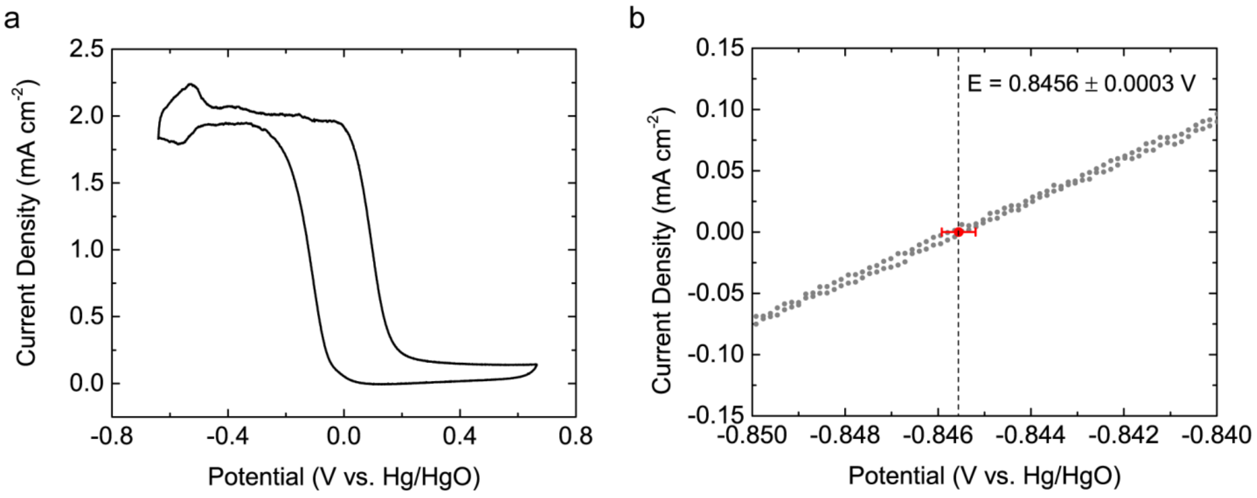

Supplementary Figure 12: Reversible hydrogen electrode (RHE) potential measurement. (**a**) Cleaning cyclic voltammogram of Pt electrode in 0.1M KOH. The electrode was cycled from +0.7V to -0.7V vs. Hg/HgO until the i-E curve stabilized in order to ensure a pristine Pt surface. The plot shown is the last cyclic voltammogram from the cleaning process. (**b**) Crossover potential for the reduction of H_2 on Pt in H_2 saturated (1 atm) 0.1M KOH. The RHE potential was determined from triplicate measurements as $E_{RHE} = E_{Hg/HgO} + (0.8456 \pm 0.0003)$ V.

Supplementary Table 1: Lattice parameters of the LCO, LSCO and SCO samples, weight fraction of Co_3O_4 and reliability factors after Rietveld refinement from PXRD data. For the samples with x = 0 – 0.4 the *R*-3*c* model was applied instead of the orbital-ordered monoclinic *I*2/*a* structure because detection of this weak distortion is beyond the resolution of our PXRD experiment.[16–18] In spite of the tetragonal superstructure detected by SAED in the x = 0.8 sample, the refinement has been performed with the cubic *Pm*-3*m* model because neither reflection splitting nor superstructure reflections are detected in the PXRD pattern. Scherrer analysis was performed on all samples on the peak at 2θ ≈ 47°, using the following formula: $= \frac{K\lambda}{\beta cos\theta}$, where τ is the mean crystallite domain size (nm), *K* is a dimensionless shape factor (0.9 for spherical particles), λ is the X-ray wavelength (0.15418 nm), β is the line broadening at FWHM (radians), and θ is the Bragg angle (°).

x in $La_{1-x}Sr_xCoO_{3-\delta}$	Space group	*a* (Å)*	*c* (Å)	wt.% Co_3O_4	R_F, R_P	τ (nm)‡
0	*R*-3*c*	5.4418(1)	13.0922(3)	1.38(5)	0.016, 0.053	18.9
0.2	*R*-3*c*	5.4436(1)	13.1567(5)	1.89(7)	0.019, 0.074	19.7
0.4	*R*-3*c*	5.4389(1)	13.1826(6)	5.51(7)	0.022, 0.069	17.3
0.6	*Pm*-3*m*	3.8374(1)	-	7.1(1)	0.022, 0.097	17.3
0.8*	*P*4/*mmm*	3.83864(8)	2*a*	0.84(5)	0.015, 0.071	17.7
1.0**	*I*4/*mmm*	7.7220(7)	15.519(2)	not present	n/a, 0.111	15.7

* - refined as cubic *Pm*-3*m*, tetragonal a_p x a_p x $2a_p$ detected from SAED.

** - unit cell parameters refined using Le Bail procedure (see Fig. S1).

‡ - mean crystallite domain size, derived from Scherrer Analysis of the peak at 2θ ≈ 47°

Supplementary Table 2: Brunauer-Emmett-Teller (BET) N_2 adsorption surface areas for oxides in this study. The surface areas were determined using a 7-point fit of the linear region of the adsorption isotherm.

x in $La_{1-x}Sr_xCoO_{3-\delta}$	BET Surface Area ($m^2\ g^{-1}$)
0	4.0
0.2	4.5
0.4	4.2
0.6	4.3
0.8	3.1
1.0	3.6
IrO_2	14.5

Supplementary Table 3: Computed reaction enthalpy changes of both LOM and AEM on $SrCoO_3$ for the construction Figure 8d. ΔE_i is labeled in the same order as shown in Figure 8d. By comparison, we find that our results of AEM agree well with Supplementary Reference 19.[19]

	ΔE_1	ΔE_2	ΔE_3	ΔE_4
LOM	1.18	1.07	1.04	1.73
AEM	1.91	1.06	0.93	1.01
AEM in Ref. 19	1.82	1.09	0.86	1.15

Supplementary Table 4: DFT-predicted corrections for zero-point energy (eV) and vibrational entropy (eV) of adsorbates; Δ(ZPE-TS) is with respect to H_2O (l) and H_2 (g).

	ZPE	TS	ZPE-TS	Δ(ZPE-TS)
H_2O (l)	0.57	0.67	-0.10	-
H_2(g)	0.28	0.40	-0.12	-
-OH	0.34	0.11	0.23	0.27
-O	0.07	0.06	0.01	-0.01
-OOH	0.43	0.28	0.15	0.17
-OO	0.13	0.14	-0.01	-0.05
-OO with O_{vac}	0.13	0.14	-0.01	-0.03
-H on lattice O	0.32	0.10	0.22	0.28

Supplementary Table 5: The following table lists the conversion factor per material to convert between geometric current density to specific current density. In addition the total area of composite material and of pure oxide is given in the table based on a 70% mass loading of NC (1080 $m^2\ g^{-1}$)

Perovskite:	Total Electrode Mass Loading (µg)	% Perovskite	BET S.A. ($m^2\ g^{-1}$)	Conversion Factor	Total Oxide Electrode Area (cm^2)	Total Electrode Area (carbon S.A. + Oxide) (cm^2)
**LCO**	10	29.42	4	1.666	0.118	75.718
**LSCO82**	10	30.19	4.5	1.443	0.136	75.736
**LSCO64**	10	29.92	4.2	1.560	0.126	75.726
**LSCO46**	10	29.47	4.3	1.547	0.127	75.727
**LSCO28**	10	26.38	3.1	2.397	0.082	75.682
**SCO**	10	30.04	3.6	1.812	0.108	75.708

Supplementary Discussion:

Catalyst Loading:

As can be seen in Supplementary Figure 8, the role of carbon has a negligible effect on the activity of IrO_2 both in geometric current density and in specific current density, yielding activities that corroborate the activities found in 3-electrode thin film RDE testing described in the literature. However, as has been noted in a number of papers, the low conductivities of perovskites require the use of carbon additives to help wire the catalysts to the electrode surface and to provide good electrical conductivity to the oxides. We have optimized our electrode preparation to maximize the gravimetric current density by using a composite of 30 wt% perovskite supported on a number of carbons and a total mass loading of 51 ug_{total}/cm^2_{geom}. We have also tested our catalysts using the protocol described in Supplementary Reference 20 (shown in the plots as "85wt%", and have found that the high electrode mass loadings (~300 ug_{total}/cm^2_{geom}) and relatively low amount of carbon (15 wt%) detract from the material performance and therefore do not describe the "true" activities of the catalysts due to inadequate wiring of the catalysts and poor utilization of the catalysts from increased mass transfer constraints in the thicker films.[20] In addition, we use a technique of spin coating our catalysts inks to provide uniform electrode films based on a study of electrode drying techniques.[21] It should be noted that in Supplementary Figure 8, the 85wt% samples had a larger mass loading of carbon compared to the 30% mass loading samples (46 $ug/cm^2_{geom.}$ For 85wt% and 35.7 ug/cm^2_{geom} for the 30 wt% samples). Yet, the poorer catalytic activity of the 85 wt% samples confirm that carbon corrosion does not significantly influence the results of the RDE cyclic voltammetry testing of the catalysts.

A full evaluation of active surface area of the catalysts based on BET surface area measurements and mass loadings is presented in Supplementary Table 5. The following formulas are used to calculate the given quantities in the Table:

$$i_s(mA\ cm_{ox}^{-2}) = (Conversion\ Factor) \times i_{geom}\ (mA\ cm_{geom}^{-2}) \quad (1)$$

$$Conversion\ Factor = \frac{0.196\ cm^2_{geom}}{\%\ oxide \times total\ electrode\ mass\ loading\ (g) \times S.A._{BET}\ (m^2\ g^{-1}) \times 10000\ cm^2\ m^{-2}} \quad (2)$$

Extension of LOM to Other Oxides:

The basis of this mechanism focuses on the covalency of the crystalline M-O bond. The covalency of the M-O bond can be determined by the position of the O p-band center relative to the Fermi level either through spectroscopic techniques or through computational approaches.[9,22] Further, the covalency can be modulated electrochemically or through substitution of lower valence elements into the A-site which in effect tunes the position of the transition metal 3d π* band and the stoichiometry of oxygen, whereby a high vacancy concentration is indicative of a high degree of overlap between the metal 3d and oxygen O 2p bands in the crystal.[14,23,24] It has been demonstrated that the O 2p band center scales with the binding energies of the intermediates during the OER and that it also controls the surface exchange coefficient in SOFCs.[9,22] Although it should be noted that the position of the M 3d π* band and thus the covalency of the surface states is expected to be different than those in the bulk.[25] Interestingly, the requirement that precious metals, such as Pt, Ir, and Ru, require an oxide film on their surface to initiate the OER, points to the fact that crystalline lattice oxygen species are a necessary component for OER electrocatalysis in alkaline electrolytes. In addition, it cannot be overlooked that doping of lower valence ions into the A-site of perovskites to increase the covalency of the B-O bond is beneficial for all types of heterogeneous catalysis on perovskite surfaces, including both the ORR and OER, regardless of the nature of the B-site (Supplementary Figure 9).[13,23,24,26–33] The high activities of $Ba_{0.5}Sr_{0.5}Co_{0.8}Fe_{0.2}O_{3-\delta}$ ($\delta = 0.4$) as well as other layered double perovskite cobaltites can also be rationalized in terms of this vacancy mediated mechanism due to their high oxygen vacancy concentrations and high electronegativity of the active Co site.[8,9] Further, the vacancy mediated mechanism is not exclusive to perovskites nor alkaline electrolytes. Through oxygen isotope exchange measurements, lattice oxygen has been demonstrated to be involved during the OER on the precious

metal-oxides, IrO_2 and RuO_2, as well as layered-oxides, such as $LiNiO_2$, and spinel oxides, like Co_3O_4.[34–39] Therefore the covalency parameter and the vacancy content should both be taken into account in the rational design of oxygen evolution catalysts. It also consistent with previous models of OER activity scaling with d-electron filling and bulk oxide formation energies, as the covalency of the M-O bond is expected to increase with the electronegativity of the transition metal.[23,30,40,41]

Supplementary Methods:

General:

All chemicals were used as received. Anhydrous ethanol and 5 wt% nafion solution in lower alcohols were purchased from Sigma-Aldrich. Lanthanum (III) nitrate hexahydrate (99.999%), strontium (II) nitrate hexahydrate (99.9%), cobalt (II) nitrate hexahydrate (99.9%), tetrapropylammonium bromide (98%), tetramethylammonium hydroxide pentahydrate (99%), 2-propanol, potassium hydroxide, were obtained from Fisher Scientific. Absolute ethanol (200 proof) was obtained from Aaper alcohol. The commercial IrO_2 sample was obtained from Strem Chemicals Inc. Oxygen (99.999%) and argon (99.999%) gases were obtained from Praxair. Vulcan carbon XC-72 was obtained from Cabot Corporation, and the nitrogen-doped carbon was prepared as reported elsewhere.[42]

Surface Atomic Characterization:

The SCO sample was handled in an Ar-filled glove box. TEM specimen was prepared in the glove box by crushing the crystals in a mortar in anhydrous ethanol and depositing drops of suspension onto holey carbon grids. The sample was transported to the microscope column completely excluding contact with air. It was found that exposing the sample to air resulted in an amorphous surface layer likely due to the formation of $SrCO_3$. For the LCO sample, the preparation could be done in air. High angle annular dark field scanning transmission electron microscopy (HAADF-STEM) images and annular bright field STEM (ABF-STEM) images were obtained with an aberration-corrected Titan G3 electron microscope operated at 200 kV.

Calibration of Rotating Ring-Disk Electrode:

Linear sweep voltammetry was performed using a rotating ring-disk electrode (RRDE) with a glass carbon disk and a Pt ring in 0.3 mM ferrocene-methanol in 0.1M KCl. The disk potential was scanned from 0.1 to 0.6 V vs. Hg/HgO (1M KOH) while the ring was held at 0.1 V vs. Hg/HgO (1M KOH). Rotation rates of ω = 400, 800, 1200, 1600, and 2000 rpm were used.

Oxygen Diffusion Rate Measurements:

Glassy carbon rotating disk electrodes with a thin layer of perovskite/VC were initially cycled in Ar saturated 1M KOH at 20 mV s^{-1} for 2 cycles. The $E_{1/2}$ of oxygen intercalation/de-intercalation was determined from the halfway potential between the anodic and cathodic peaks. For materials with low oxygen vacancy concentration (i.e. $x \leq 0.4$) the $E_{1/2}$ was determined as the open-circuit potential. After being cycled, the oxygen ion diffusion rate was measured chronoamperometrically, using the same electrode, by applying a potential 50 mV more anodic of the $E_{1/2}$. The electrodes were spun at 1600 rpm to get rid of electrolyte based mass transfer effects, and the current was measured as a function of time for 4 hrs. The current was plotted versus $t^{-1/2}$ and the linear section of the curve was fit to find the intercept with the $t^{-1/2}$ axis.

Measurement of RHE Potential:

Initially, a stationary Pt RDE electrode (0.196 cm^2, Pine Instruments) was cleaned through cyclic voltammetry at 20 mV s^{-1} from +0.7V to -0.7V vs. Hg/HgO (1M KOH) in Ar saturated 0.1M KOH in a 3-electrode cell configuration using a Pt wire counter electrode and a Hg/HgO (1M KOH) reference electrode. The cell was then saturated with H_2 gas (1 atm) and the reduction/oxidation of hydrogen was measured through cyclic voltammetry at 1 mV s^{-1} rotating the electrode at ω=1600 rpm. The potential at which $i = 0$ was measured for 3 separate scans, ensuring saturation conditions of H_2 between each scan.

Density Function Theory Calculations and Surface Models:[6,19,43–45]

Spin polarized calculations are performed using VASP with PAW pseudopotentials and the RPBE-GGA functional. An energy cutoff of 500 eV and a 2x2x1 *k*-point mesh are used for all calculations. Symmetric supercells are composed of 7-atomic-layer-thick slabs separated by ~20 Å of vacuum perpendicular to the surface to prevent spurious interactions due to periodic boundary conditions. A (2×2) in-plane supercell is employed. The systems are initiated with the Co atoms in a ferromagnetic configuration, which is allowed to evolve during the electronic optimization. Structures are relaxed until

the forces on each atom are less than 10^{-4} eV/Å. Free energies are computed directly from DFT by approximating the free energy of solids by the DFT total energy, and computing corrections for the zero-point energy and vibrational entropy for gas phase species and surface adsorbates (Supplementary Table 4).We note that tests with GGA+*U* and various U values result in weaker adsorption strength of –O (I_0 in Figure 7, Main Text), further increasing the relative stability of I_1 to I_0 and thus do not qualitatively change our results.

We consider [001] surfaces, as these are observed experimentally. Along the (001) direction, LSCO consists of alternating LaO (with substituted Sr) and CoO_2 atomic planes, with nominal charges of +1 and -1, respectively. Both polar and non-polar slabs can therefore be constructed. However, as polar fields are energetically costly, they are likely compensated in realistic catalyst systems; thus we consider only non-polar slabs. Surfaces are initially built based on the stoichiometric cubic phase of LSCO (Supplementary Figure 8a). We note that LSCO is observed to undergo phase changes with increased oxygen deficiency. Thus, the rhombohedral $LaCoO_3$ and the $SrCoO_{2.75}$ are also investigated (Supplementary Figure 8b-d). We find that neither the phase nor the bulk oxygen stoichiometry changes the relative stability of I_1 to I_0 for the whole range of LSCO compositions.

To elucidate the lattice-oxygen-participated (LOM) OER mechanism on LSCO, we begin by comparing the free energy (stability) of each adsorbate-evolution-mechanism (AEM) intermediate (adsorbed -O, -OH, -OO or -OOH) with various isomers, in which lattice O combines with each AEM adsorbate, forming a surface O vacancy. As the surface oxygens of LSCO may be partially protonated to form lattice hydroxides, it is thus possible to have two kinds of isomeric surfaces for a given AEM intermediate, with adsorbates either originating from (i) the unprotonated surface O or (ii) the lattice hydroxides. For example, the adsorbed –O (AEM) could have two possible isomeric configurations: (i) adsorbed –OO with surface O vacancy, and (ii) adsorbed –OOH with surface O vacancy, where the H and one of the oxygens in the –OOH come from a lattice hydroxide. Our calculations show that only the

configuration with the adsorbed –OO and surface O vacancy (I_1), which is the isomeric intermediate of adsorbed –O (I_0), is more energetically favorable than the corresponding AEM intermediate (Figure 7a, Main Text).

Supplementary References:

1. Van Buren, F. R., Broers, G. H. J., Bouman, A. J. & Boesveld, C. An electrochemical method for the determination of oxygen ion diffusion coefficients in $La_{1-x}Sr_xCoO_{3-y}$ compounds: Theoretical aspects. *J. Electroanal. Chem. Interfacial Electrochem.* **87,** 389–394 (1978).
2. Van Buren, F. R., Broers, G. H. J., Bouman, A. J. & Boesveld, C. The electrochemical determination of oxygen ion diffusion coefficients in $La_{0.50}Sr_{0.50}CoO_{3-y}$: Experimental results and related properties. *J. Electroanal. Chem. Interfacial Electrochem.* **88,** 353–361 (1978).
3. Kobussen, A. G. C., van Buren, F. R. & Broers, G. H. J. The influence of the particle size distribution on the measurement of oxygen ion diffusion coefficients in $La_{0.50}Sr_{0.50}CoO_{3-y}$. *J. Electroanal. Chem. Interfacial Electrochem.* **91,** 211–217 (1978).
4. Radaelli, P. G. & Cheong, S.-W. Structural phenomena associated with the spin-state transition in $LaCoO_3$. *Phys. Rev. B* **66,** 094408 (2002).
5. Wagman, D. D. *et al. Selected values of chemical thermodynamic properties. Part 1. Tables for the first 23 elements in the standard order of arrangement*. (National Bureau of Standards, 1965). at <http://archive.org/details/selectedvaluesof2701wagm>
6. Rong, X. & Kolpak, A. M. Ab Initio Approach for Prediction of Oxide Surface Structure, Stoichiometry, and Electrocatalytic Activity in Aqueous Solution. *J. Phys. Chem. Lett.* **6,** 1785–1789 (2015).
7. Bockris, J. O. & Otagawa, T. Mechanism of oxygen evolution on perovskites. *J. Phys. Chem.* **87,** 2960–2971 (1983).
8. Suntivich, J., May, K. J., Gasteiger, H. A., Goodenough, J. B. & Shao-Horn, Y. A Perovskite Oxide Optimized for Oxygen Evolution Catalysis from Molecular Orbital Principles. *Science* **334,** 1383–1385 (2011).
9. Grimaud, A. *et al.* Double perovskites as a family of highly active catalysts for oxygen evolution in alkaline solution. *Nat. Commun.* **4:2439,** (2013).

10. Hardin, W. G. *et al.* Tuning the Electrocatalytic Activity of Perovskites through Active Site Variation and Support Interactions. *Chem. Mater.* **26,** 3368–3376 (2014).

11. Wattiaux, A., Grenier, J. C., Pouchard, M. & Hagenmuller, P. Electrolytic oxygen evolution in alkaline medium on $LaSrFeO_3$ perovskite-related ferrites. *J. Electrochem. Soc.* **134,** 1714–1718 (1987).

12. Matsumoto, Y. & Sato, E. Oxygen evolution on $La_{1-x}Sr_xMnO_3$ electrodes in alkaline solutions. *Electrochimica Acta* **24,** 421–423 (1979).

13. Kim, J., Yin, X., Tsao, K.-C., Fang, S. & Yang, H. $Ca_2Mn_2O_5$ as Oxygen-Deficient Perovskite Electrocatalyst for Oxygen Evolution Reaction. *J. Am. Chem. Soc.* **136,** 14646-14649 (2014).

14. Hong, W. T. *et al.* Probing $LaMO_3$ Metal and Oxygen Partial Density of States Using X-ray Emission, Absorption, and Photoelectron Spectroscopy. *J. Phys. Chem. C* **119,** 2063–2072 (2015).

15. Gorlin, Y. & Jaramillo, T. F. A Bifunctional Nonprecious Metal Catalyst for Oxygen Reduction and Water Oxidation. *J. Am. Chem. Soc.* **132,** 13612–13614 (2010).

16. Maris, G. *et al.* Evidence for orbital ordering in $LaCoO_3$. *Phys. Rev. B* **67,** 224423 (2003).

17. Takami, T., Zhou, J.-S., Goodenough, J. B. & Ikuta, H. Correlation between the structure and the spin state in $R_{1-x}Sr_xCoO_3$ (R = La, Pr, and Nd). *Phys. Rev. B* **76,** 144116 (2007).

18. Wang, Y. *et al.* Correlation between the Structural Distortions and Thermoelectric Characteristics in $La_{1-x}A_xCoO_3$ (A = Ca and Sr). *Inorg. Chem.* **49,** 3216–3223 (2010).

19. Hammer, B., Hansen, L. B. & Nørskov, J. K. Improved adsorption energetics within density-functional theory using revised Perdew-Burke-Ernzerhof functionals. *Phys. Rev. B* **59,** 7413–7421 (1999).

20. Suntivich, J., Gasteiger, H. A., Yabuuchi, N. & Shao-Horn, Y. Electrocatalytic Measurement Methodology of Oxide Catalysts Using a Thin-Film Rotating Disk Electrode. *J. Electrochem. Soc.* **157,** B1263–B1268 (2010).

21. Garsany, Y., Singer, I. L. & Swider-Lyons, K. E. Impact of film drying procedures on RDE characterization of Pt/VC electrocatalysts. *J. Electroanal. Chem.* **662,** 396–406 (2011).

22. Lee, Y.-L., Kleis, J., Rossmeisl, J., Shao-Horn, Y. & Morgan, D. Prediction of solid oxide fuel cell cathode activity with first-principles descriptors. *Energy Environ. Sci.* **4,** 3966–3970 (2011).

23. Fujimori, A. Electronic structure of metallic oxides: Band-gap closure and valence control. *J. Phys. Chem. Solids* **53,** 1595–1602 (1992).

24. Du, J. *et al.* Nonstoichiometric Perovskite $CaMnO_{3-\delta}$ for Oxygen Electrocatalysis with High Activity. *Inorg. Chem.* **53,** 9106–9114 (2014).

25. Eglitis, R. I. Ab initio calculations of $SrTiO_3$, $BaTiO_3$, $PbTiO_3$, $CaTiO_3$, $SrZrO_3$, $PbZrO_3$ and $BaZrO_3$ (001), (011) and (111) surfaces as well as F centers, polarons, KTN solid solutions and Nb impurities therein. *Int. J. Mod. Phys. B* **28,** 1430009 (2014).

26. Peña, M. a & Fierro, J. L. Chemical structures and performance of perovskite oxides. *Chem. Rev.* **101,** 1981–2017 (2001).

27. Choi, S. O., Penninger, M., Kim, C. H., Schneider, W. F. & Thompson, L. T. Experimental and Computational Investigation of Effect of Sr on NO Oxidation and Oxygen Exchange for $La_{1-x}Sr_xCoO_3$ Perovskite Catalysts. *ACS Catal.* **3,** 2719–2728 (2013).

28. Fierro, C., Carbonio, R. E., Scherson, D. & Yeager, E. B. In situ mossbauer effect spectroscopy of a model iron perovskite electrocatalyst. *Electrochimica Acta* **33,** 941–945 (1988).

29. Kovalevsky, A. V., Sviridov, D. V., Kharton, V. V., Naumovich, E. N. & Frade, J. R. Oxygen Evolution on Perovskite-Type Cobaltite Anodes: An Assessment of Materials Science-Related Aspects. *Mater. Sci. Forum* **514-516,** 377–381 (2006).

30. Calle-Vallejo, F., Diaz-Morales, O., Kolb, M. & Koper, M. T. M. Why is bulk thermochemistry a good descriptor for the electrocatalytic activity of transition metal oxides? *ACS Catal.* **5**, 8698-873 (2014).

31. Matsumoto, Y., Yamada, S., Nishida, T. & Sato, E. Oxygen Evolution on $La_{1-x}Sr_xFe_{1-y}Co_yO_3$ Series Oxides. *J. Electrochem. Soc.* **127,** 2360–2364 (1980).

32. Meadowcroft, D. B. Low-cost Oxygen Electrode Material. *Nature* **226,** 847–848 (1970).

33. Wu, N.-L., Liu, W.-R. & Su, S.-J. Effect of oxygenation on electrocatalysis of $La_{0.6}Ca_{0.4}CoO_{3-x}$ in bifunctional air electrode. *Electrochimica Acta* **48,** 1567–1571 (2003).

34. Fierro, S., Nagel, T., Baltruschat, H. & Comninellis, C. Investigation of the oxygen evolution reaction on Ti/IrO_2 electrodes using isotope labelling and on-line mass spectrometry. *Electrochem. Commun.* **9,** 1969–1974 (2007).

35. Burke, L. D., Murphy, O. J., O'Neill, J. F. & Venkatesan, S. The oxygen electrode. Part 8.—Oxygen evolution at ruthenium dioxide anodes. *J. Chem. Soc. Faraday Trans. 1 Phys. Chem. Condens. Phases* **73,** 1659–1671 (1977).

36. Wohlfahrt-Mehrens, M. & Heitbaum, J. Oxygen evolution on Ru and RuO_2 electrodes studied using isotope labelling and on-line mass spectrometry. *J. Electroanal. Chem. Interfacial Electrochem.* **237,** 251–260 (1987).

37. C. Lee, A. Riga & E. Yeager. *Mass Transport Phenomena in Ceramics*. (Plenum Press, 1975).

38. Arunkumar, T. A., Wu, Y. & Manthiram, A. Factors Influencing the Irreversible Oxygen Loss and Reversible Capacity in Layered $Li[Li_{1/3}Mn_{2/3}]O_2$–$Li[M]O_2$ ($M = Mn_{0.5-y}Ni_{0.5-y}Co_{2y}$ and $Ni_{1-y}Co_y$) Solid Solutions. *Chem. Mater.* **19,** 3067–3073 (2007).

39. Xiao, J. *et al.* Surface Structure Dependent Electrocatalytic Activity of Co_3O_4 Anchored on Graphene Sheets toward Oxygen Reduction Reaction. *Sci. Rep.* **3,** (2013).

40. Vojvodic, A. & Nørskov, J. K. Optimizing Perovskites for the Water-Splitting Reaction. *Science* **334,** 1355–1356 (2011).

41. Man, I. C. *et al.* Universality in Oxygen Evolution Electrocatalysis on Oxide Surfaces. *ChemCatChem* **3,** 1159–1165 (2011).

42. Wang, X. *et al.* Ammonia-Treated Ordered Mesoporous Carbons as Catalytic Materials for Oxygen Reduction Reaction. *Chem. Mater.* **22,** 2178–2180 (2010).

43. Kresse, G. & Furthmüller, J. Efficient iterative schemes for *ab initio* total-energy calculations using a plane-wave basis set. *Phys. Rev. B* **54,** 11169–11186 (1996).

44. Blöchl, P. E. Projector augmented-wave method. *Phys. Rev. B* **50,** 17953–17979 (1994).

45. Rong, X., Parolin, J. & Kolpak, A. M. A Fundamental Relationship between Reaction Mechanism and Stability in Metal Oxide Catalysts for Oxygen Evolution. *ACS Catal.* **6,** 1153–1158 (2016).
